# A simplified interventional mapping system (SIMS) for the selection of combinations of targeted treatments in non-small cell lung cancer

**DOI:** 10.18632/oncotarget.3741

**Published:** 2015-04-03

**Authors:** Vladimir Lazar, Eitan Rubin, Stephane Depil, Yudi Pawitan, Jean-François Martini, Jesus Gomez-Navarro, Antoine Yver, Zhengyin Kan, Jonathan R. Dry, Jeanne Kehren, Pierre Validire, Jordi Rodon, Philippe Vielh, Michel Ducreux, Susan Galbraith, Manfred Lehnert, Amir Onn, Raanan Berger, Marco A. Pierotti, Angel Porgador, CS Pramesh, Ding-wei Ye, Andre L. Carvalho, Gerald Batist, Thierry Le Chevalier, Philippe Morice, Benjamin Besse, Gilles Vassal, Andrew Mortlock, Johan Hansson, Ioana Berindan-Neagoe, Robert Dann, Joel Haspel, Alexandru Irimie, Steve Laderman, Hovav Nechushtan, Amal S. Al Omari, Trent Haywood, Catherine Bresson, Khee Chee Soo, Iman Osman, Hilario Mata, Jack J. Lee, Komal Jhaveri, Guillaume Meurice, Gary Palmer, Ludovic Lacroix, Serge Koscielny, Karina Agda Eterovic, Jean-Yves Blay, Richard Buller, Alexander Eggermont, Richard L. Schilsky, John Mendelsohn, Jean-Charles Soria, Mace Rothenberg, Jean-Yves Scoazec, Waun Ki Hong, Razelle Kurzrock

**Affiliations:** ^1^ Gustave-Roussy Cancer Center, Villejuif, France; ^2^ WIN Consortium, Villejuif, France; ^3^ Ben-Gurion University of the Negev, Beer-Sheva, Israel; ^4^ Leon Berard Cancer Center, Lyon, France; ^5^ Karolinska Institutet, Stockholm, Sweden; ^6^ Pfizer Oncology Research, San Diego, CA; ^7^ Pfizer Oncology, Pfizer Inc, New York, NY, USA; ^8^ Takeda Pharmaceuticals International Co., Cambridge, MA, USA; ^9^ AstraZeneca Pharmaceuticals LP, Global Medicines Development, Gaithersburg MD, USA; ^10^ Oncology iMED, Waltham, MA, USA; ^11^ Oncology iMED, Macclesfield, Cheshire UK; ^12^ Sanofi, Paris, France; ^13^ Institut Mutualiste Montsouris, Paris, France; ^14^ Vall d'Hebron Institute of Oncology Universitat Autonoma de Barcelona, Barcelona, Spain; ^15^ University Paris-Sud, Kremlin-Bicetre, France; ^16^ Chaim Sheba Medical Center, Tel-Hashomer, Israel; ^17^ Fondazione IRCCS Istituto Nazionale dei Tumori, Milano, Italy; ^18^ Tata Memorial Centre, Mumbai, India; ^19^ Fudan University Shanghai Cancer Center, Shanghai, China; ^20^ Fundacao Pio XII – Barretos Cancer Hospital, Barretos, Brazil; ^21^ Segal Cancer Centre at the Jewish General Hospital, McGill University, Montreal, QC, Canada; ^22^ University of Medicine and Pharmacy Iuliu Hatieganu, Cluj-Napoca, Romania; ^23^ Ion Chiricuta Oncology Institut, Cluj-Napoca, Romania; ^24^ General Electric Healthcare, Westborough, MA, USA; ^25^ Oracle Corporation, Reading, UK; ^26^ Agilent Technologies, Santa Clara, CA, USA; ^27^ Hadassah Hebrew University Medical Center, Jerusalem, Israel; ^28^ King Hussein Cancer Center, Amman, Jordan; ^29^ Blue Cross Blue Shield Association, Chicago, IL, USA; ^30^ National Cancer Centre, Singapore; ^31^ New York University Langone Medical Center, NY, USA; ^32^ The University of Texas MD Anderson Cancer Center, Houston, Texas, USA; ^33^ Foundation Medicine Inc., Cambridge, MA, USA; ^34^ American Society of Clinical Oncology (ASCO), Alexandria, VA, USA; ^35^ UC San Diego - Moores Cancer Center, La Jolla, CA, USA

**Keywords:** Tri-therapy, NSCLC, targeted therapies, algorithm, pathway

## Abstract

Non-small cell lung cancer (NSCLC) is a leading cause of death worldwide. Targeted monotherapies produce high regression rates, albeit for limited patient subgroups, who inevitably succumb. We present a novel strategy for identifying customized combinations of triplets of targeted agents, utilizing a simplified interventional mapping system (SIMS) that merges knowledge about existent drugs and their impact on the hallmarks of cancer. Based on interrogation of matched lung tumor and normal tissue using targeted genomic sequencing, copy number variation, transcriptomics, and miRNA expression, the activation status of 24 interventional nodes was elucidated. An algorithm was developed to create a scoring system that enables ranking of the activated interventional nodes for each patient. Based on the trends of co-activation at interventional points, combinations of drug triplets were defined in order to overcome resistance. This methodology will inform a prospective trial to be conducted by the WIN consortium, aiming to significantly impact survival in metastatic NSCLC and other malignancies.

## INTRODUCTION

Lung cancer is one of the most prevalent and deadly malignancies, contributing a staggering 1.6 million cases diagnosed per year and about 21% of cancer deaths to the global cancer burden [1, 2]. The majority of individuals with non-small cell lung cancer (NSCLC) present at the metastatic stage, and most patients with localized disease will relapse. The standard of care in advanced disease- mainly cytotoxic chemotherapy and targeted agents for selected subsets, has had modest impact on mortality, with dismal one- and five-year survival rates of around 15% and 4%, respectively, for patients with metastatic disease [1, 2]. For patients who have failed first-line therapy, the median survival is only about seven months.

Targeted therapies implemented in standard care are directed at the activated products of mutated epidermal growth factor receptor (EGFR) [3] or ALK translocation [4], and have shown high response rates, and improved progression-free survival (PFS). However, these monotherapies apply to only small subsets of oncogene-driven patients, and virtually all develop resistance and succumb to their disease [5]. Relapse occurs mostly as a consequence of the Darwinian selection of tumour clones harboring genomic variants that lead to the activation of additional signaling pathways and, hence, resistance. This is perhaps not unexpected, as tumors often exhibit a large variety of molecular abnormalities [6, 7], even at diagnosis. Heterogeneity across tumor clones is amplified in metastases, and in response to therapeutic pressure.

Combination of cytotoxic therapies have been demonstrated to be effective where single agents provide only moderate benefits, as illustrated in Hodgkin's lymphoma. Whether this paradigm applies to targeted therapy remains unclear for most diseases. However, recently, combinations targeting the same pathway (e.g. trametanib (MEK) inhibitor together with dabrafenib (BRAF inhibitor) in *BRAF*-mutant melanoma [8] or resistance pathways (combining PIK3CA and MEK inhibitors) [9] showed efficacy, either preclinically and/or in the clinic. With the aim of further enhancing clinical benefit and increasing survival, we intend to explore the efficacy of triple regimen therapy, following the historical success in diseases such as acquired immunodeficiency syndrome. The major challenges for this effort are delineating the scientific rationale for matching agents with patients' tumors, while being cognizant of potential toxicity for multi-drug regimens.

The nosologic segmentation of NSCLC based mainly on genomics heralded a new era, enabling successful development of targeted monotherapy for selected molecular subsets [3, 4, 10, 11]. The recent report of the Cancer Genome ATLAS Research Network shows that 62% of lung adenocarcinomas harbored activating mutations in known driver oncogenes; cancer-associated mutations in *KRAS* (32%), *EGFR* (11%) and *BRAF* (7%), were common [12]. Despite this progress, many patients still have no identified druggable genomic alterations and, as previously mentioned, most of those who do rapidly relapse. Strategies to delineate rational combinations of targeted therapy, using multi-platform, advanced omics technologies that move beyond genomics alone in NSCLC are lacking. Using novel tools and paradigms, the complexity of molecular aberrations in cancer may be better understood in terms of critical convergence pathways. In the present report, we propose a pragmatic approach using a simplified interventional points matching system (SIMS) that will produce customized triple therapy regimens for individual patients based on the most common abnormalities found in a genomic/transcriptomic analysis of matched tumor and normal biopsies from 121 patients with lung cancer.

## RESULTS

### Overview of the Simplified Interventional Points Matching System (SIMS) strategy

Our objective was to establish a realistic framework that would allow useful drug combinations to be identified in a personalized way (i.e. matching the combination to the patient based on the tumor characteristics). This strategy involved three steps: *(i)* find interventional points/ nodes/ markers for common classes of drugs. (We delineated 24 markers covering 183 genes (Table 1 and Supplemental Tables 1-7); *(ii)* find a score that summarizes the behavior of these markers in a given patient. The score should be proportional to the probability that the cognate drug(s) would produce salutary effects; and *(iii)* delineate a set number of triple drug combinations that could be tested clinically, and that would maximize the number of patients whose activated interventional nodes would be impacted.

**Table 1:** Summary of the interventional points or nodes (N=24) defined by the genes involved (N = 183) and examples of drugs that can impact these nodes

Nodes	Components of the inteventional points	Examples of drugs acting on interventional points
HER	EGF, TGFA, AREG, EREG, HUGE, BTC, NRG1, NRG2, NRG4, EGFR, ERBB2, ERBB3, ERBB4	Afatinib, Dacomitinib-(Pan-Her inhibitor)
CDK4, 6	CDK4, CDK6, CCND1, CCND2, CCND3, COKN2A, CDKN2B, CCNE1, CCNE2, CCNE3, RB1	Palbociclib (CDK4,6 inhibitor)
PLK/ AURK	PLK1, AURKA, BORA ILK, KIF11	Aurora A Kinase inhibitor
Angio genes	VEGFA, VEGFB, VEGFC, VEGFD, VEGFR1, VEGFR2, VEGFR3, PDGFA, PDGFB, PDGFRA, PDGFRB, Kit	Axltinib Motesanib
Angio poietins	THBS1, TGFBI, ANGPT1, ANGPT2, ANGPTL1, ANGPT4, TIE1, TEK	-
Immune modulator	PD1L, PDCD1LG2, PDCD1, CTLA4, LAG3	Ipilimumab (CTLA4); Tremelimumab (CTLA4), Nivolumab (PD1); AMP514 (PD1), Pidilizumab (PD-1); MED14736 (PD-L1) PF-05082566 (4-1 BB)
PI3K	PIK3CA, PIK3CB, PIK3CD, PIK3CG, PIK3C2B, PRKCB, PRKCA, PRKCB, PIK3R1, PIK3R2, PIK3R3	PF-384 (P13X/mTOR-inhibitor) AZD8186 (PI3Kb) PI3Kalpha inhibitor
MET	HGF, MET, AXL, MST1R	Crizotinib, Cabozantinib, Volitinib (cMet)
MEK	MAP2K1, MAP2K2, MAP2K3, MAP2K4, MAP3K1, MAP3K2, MAP3K3, MAP3K4	Trametanib Selumetinib (MEK)
ERK	MAPK3, MAPK1, KSR1, MAPK11	-
Antiapoptosis	BCL2, BCLXL, BIRC5, XIAP, BAk, TP53	ABT-199 (BCL-2) MK-1775 (Wee-1 inhibitor; p53)
FGF	FGF1 to FGF18, FGFR1, FGFR2, FGFR3, FGFR4	Lenvatinib, Lucitanib AZD4547 (FGFR1, 2, 3)
mTOR	mTor, AKT1, AKT2, PTEN, TSC1, TSC2, STK11, PIM1, PIM2, PIM3	Everolimus, Temsirolimus PF-384 (P13K/mTOR inhibitor) AZD2014 (TOR kinase); AZD5363 (AKT1, 2, 3) AZD1208 (PIM1, 2); TORC1/TORC2 inhibitor
Ras/Raf	KRAS, NRAS, HRAS, RAF1, BRAF, CRAF	Trametinib, Vemurafenib, Dabrafenib Pan-RAF inhibitor
Telomerase	TERT, TERC, TEPI, HSP9OAA1, DKC1, PTGES3	-
IGF	IGF1, IGF2, IGF1R, IGF2R, INSR, IRS1, PKM	Cixitumumab Med1-573 (IGF)
Wnt	CDH1, CTNNA1, CTNNB1, WNT1, FZD1, WNT5A, B, FZDS, WIF1, DKK1	PRI-274
PARP	PARP1, BRCA1, XRCC1, RAD54L, RAD54B, ATM, ATR, CHEK1, CHEK2, WEE1	Olaparib (PARP) AZD1775 (Wee1) AZD6738 (ATR)
HDAC	HDAC1, HDAC2, HDAC3, HDAC4, HDACS	Vorinostat
JAK-STAT	JAK1, JAK2, STAT1, STAT2, STAT3, SOCS1	Riluxitinib; AZD9150
Hedgehog	SHH, PTCH1, SMO, STK36, PRKACA, SUFU,	Vismodegib
NOTCH	NOTCH1, Adam17, PSEN1, NCSTN, JAG1, SRRT, APH1A	LY3039478
DNA Repair	ERCC1, FtAD52, XRCC4, RAD51, BRCA1, NEDD8, NAE1	NEDD8 activating enzyme inhibitor
Others	RET, ALK, ROS1, UB1	Crizotinib, Ceritinib, Sorafenib, Cabozantinib

Interventional points are defined by genes/group of genes that, when activated, could be blocked by a customized therapy combination.

**Figure 1:**
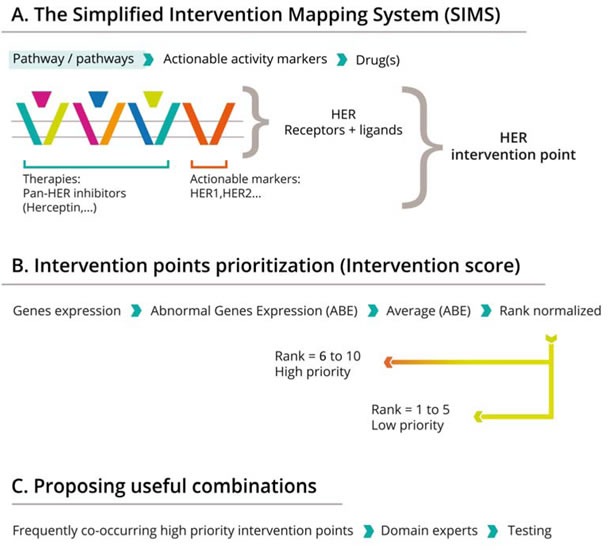
The framework for combinatorial personalized cancer medicine The SIMS strategy has three steps: **A**: Mapping therapeutic efficacy to cellular components and identification of interventional nodes. The example outlines the HER interventional point, constituted by four receptors (EGFR, Her2, Her3 and Her4) and their major ligands (EGF, TGFA, NRG1, NRG2, NRG4 and NRG4). Activation of this node can be induced by receptor mutations or overexpression of receptors and or ligands in tumor as compared with the normal counterpart, and this activation can be efficiently blocked by a panHer inhibitor, such as a fatinib; **B**: Scoring the status of specific nodes in the interventional maps defined, and predicting combination efficacy. Interventional points scored over 5 (6 to 10) are high priority. C. Finding the most frequent co-existing interventional nodes and hence suggesting combinations. Frequently co-occurring, high priority interventional points are determined, and cognate drugs are identified based on literature reviews.

Based on these assumptions, we proposed the SIMS (simplified interventional points mapping system) framework for precision combinational cancer medicine (Figure 1).

First, we reduced the enormous complexity of biological pathways and signaling cross talk by devising a simplified map that concentrates only on the genes that are most indicative of drug/target status. We defined “intervention points,” which consist of drug targets or groups of targets, as well as genes upstream of the targets, that together reflect a specific biological activity that is actionable through therapeutic interventions. For example, pan-HER therapies define the HER group of receptors and their ligands as a single intervention point (Figure 1A).

The second part of the work proposed a simplified approach for prioritizing intervention points for a specific patient. The basic premise behind the proposed score is that when the genes associated with an intervention point are more “disturbed” as compared to their status in normal cells (in terms of sequence and/or expression level), the more likely it is that this intervention point will be crucial to the tumor. From this it follows that the more disturbed the genes of an intervention point, the higher the probability that therapeutics targeted at that point will impact the viability of tumor cells, and hence benefit the patient.

In the present work, we first developed a family of simple scores that combine somatic mutations found in the intervention point genes with copy number variations (CNVs) and expression changes in protein-coding transcripts and in miRNAs. The rank normalization (in our example, using deciles) is used to make the scores of different intervention points comparable. We evaluated these scores *in silico* on our 121 patient NSCLC dataset.

Finally, a method is needed for integrating the scores and choosing combinations that are likely to benefit the patients. Here we used an algorithmic approach. We described the status of 24 intervention points in a panel of 121 patients with lung cancer as an example. From this foundation, we applied a knowledge-driven strategy to look for three-drug combinations that might complementarily or synergistically benefit the patient. We identified those pathways that co-occur frequently in the patients and are mechanistically independent. To further improve the efficacy of the proposed combinations, we propose to add immunomodulating therapies (i.e. anti-PD1L or anti-CTLA4) to the triplet regimens, with the additional aim of reducing the chance of drug/drug interactions and side effects (from combining, for instance, three tyrosine kinase inhibitors), while maintaining/enhancing predicted efficacy.

### Scoring of integrated genomic/transcriptomic data

After processing of the genomic data, a score was generated for all the 24 interventional points as shown in Supplemental Table 5. While somatic mutations automatically generated a score of 10, only a subset of tumors carried activating mutations. Most of the scores were obtained based on gene expression and penalized by miRNA expression. miRNA induced a significant penalty of scores for mTor, AKT, PTEN, RAS, ERK, PI3K and surprisingly PDL1, whereas impact on other interventional points was not significant. In this data set, CNV also had a non -significant impact on the score.

Assuming that preferred combinations will include two targeted therapies and an immunomodulator to attenuate risk of toxicity, we investigated the frequency of activation of PDL1 and CTLA4 (Table 2). PDL1 is activated in 63 (out of 121 patients), CTLA4 is activated in 58 (out of 121 patients) and PDL1 and CTLA4 are co-activated in 36 patients out of 121. In total 87 patients (out of 121) (71%) have one of two immune-related targets activated (PDL1 or CTLA4), whereas 36 patients (of 121) do not have activation of immune targets.

**Table 2:** The frequencies of activation of actionable interventional points in three groups of NSCLC patients

**Group 1**	**NSCLC patients with activated PD1L - 63 out of 121 NSCLC (52%)**																
No. Patients	36	63	35	30	28	27	25	28	28	31	32	23	21	51	27	29	42
% group1	30	100	56	48	44	43	40	44	44	49	51	37	33	81	43	46	67
**Group 2**	**NSCLC with activated CTLA4 - 58 out of 121 NSCLC (48%)**																
No. Patients	58	34	32	28	32	22	33	30	34	37	32	20	25	45	17	32	40
% group 2	100	59	55	48	55	38	57	52	59	64	55	34	43	78	29	55	69
**Group 3**	**NSCLC without activated PD1L or CTLA4 - 36 out of 121 NSCLC (30%)**																
No. Patients	0	0	8	19	15	17	10	18	17	20	12	14	18	19	10	17	24
% group 3	0	0	22	53	42	47	28	50	47	56	33	39	50	53	28	47	67
Activated Nodes	CTLA4	PD1L	MEK	mTOR	PI3K	ERK	MET	AURKA	CDK4,6	HER	Angio	FGF	PARP	Ras/RAF	IGF	DNA REPAIR	mTOR/PI3K

### Interventional node activation/co-activation

**Figure 2:**
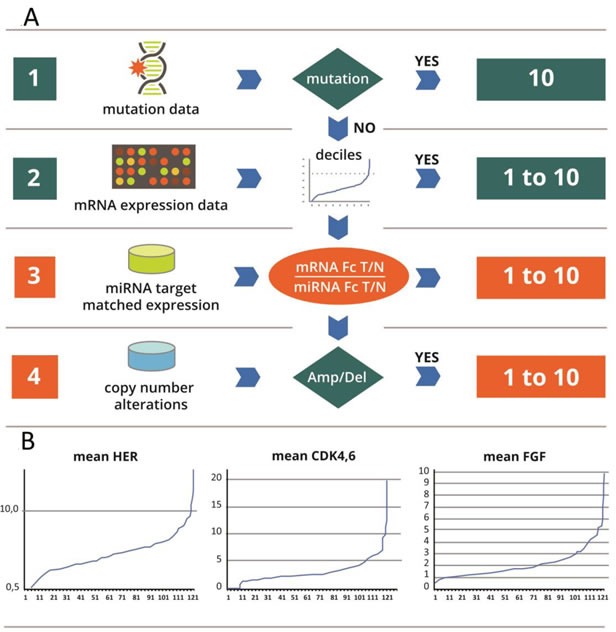
**A**: It ranges from 1 to 10. The score combines evidence from 3 data sources: mutations, meanfold change in gene over expression (mRNA and miRNA) in the tumor versus normal and copy number variation. Different data sources will trigger different weights in the score: i) activating mutations (e.g. *KRAS* in the RAS path) have decisive weight. The maximal score of 10 is given to every node with an activating mutation; ii) in the absence of a mutation, the score is based on weighted sum of the mRNA meanfold changes corrected by an adjustment based on miRNAs and to a lesser extent on CNV abnormalities. **B**: Principle of the calibrator: In Y: Fold change (Fc) of differential gene expression between tumor (T) and normal (N) in each patient. In X: number of patients (No): for each graph, the order of patients is different. This series serves as a calibrator for calculation of deciles. For every new measurement in each patient, the meanfold change for mRNA is plotted against the calibrator curve, and the deciles partition of the curve enables assignment of a score from 1 to 10. The score obtained based on the mRNA is corrected by miRNA, and is considered in the absence of mutations.

In the next step, we made the selection of all activated interventional points. Scores 8, 9 and 10 were designated high activation, whereas scores 6 and 7 were designated medium activation. Scores <6 were designated as non-activated interventional points. This threshold was determined based on the distribution of values for each interventional point in the data set of 121 patients. Figure 2B shows this distribution for three nodes; however all the other points had a similar trajectory.

**Figure 3:**
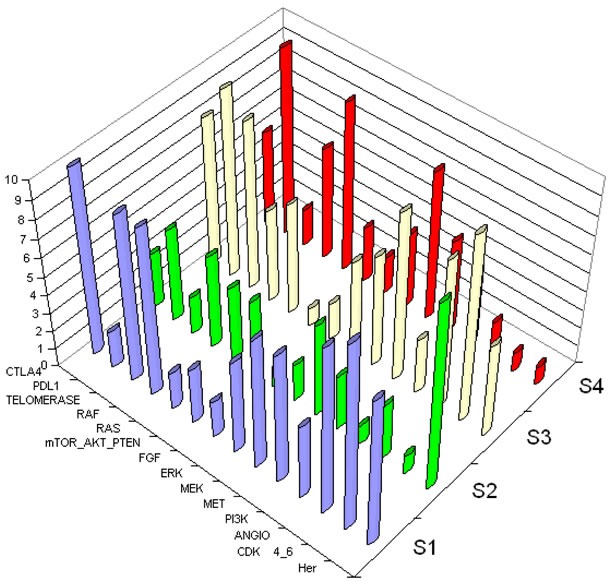
3D representation of the scoring system Axis Z shows score from 1 to 10 of each interventional point. Axis X represents examples of interventional points. Axis Y represents each patient. Four subjects are shown to demonstrate the complexity of co-activation of interventional points. Abbreviations used to designate interventional points are described in Table 1. Each patient's tumor shows numerous activations, suggesting multiple possibilities for combinations. S1, S2, S3, and S4 each represent an individual patient.

Supplemental Tables 6 and 7 show trends of co-activations of the 24 interventional points, and provide new insights into the biology of NSCLC, demonstrating the complexity of co-activation of interventional points. Figure 3 illustrates that each patient has a large number of activated nodes, and thus will have multiple therapeutic choices. Overall, the most commonly activated interventional points in all 121 patients were CDK4/6, Ras/Raf, anti-apoptosis, Her, Notch and Polokinase-Aurora-Kinase.

Activation of immunomodulator-related interventional nodes appears to be relevant to over half of NSCLC. Focusing on prevalence of activation of PDL1 and CTLA4, we analyzed specifically the frequency of activation of the other interventional points, as shown in Tables 2 and 3. Interestingly, RAS and RAF nodes are co-activated in a majority of patients, as are mTOR and PI3K.

**Table 3:** Summary of the most frequent triple combinations and summary of the most frequent combinations involving the PD1L immunomodulator (Bolded rows indicate the six most frequent combinations involving the PD1L immunomodulator)

First drug	No.	Second drug	No.	Third drug	No.	%
RAS/RAF	88	mTor/P13K	60	PD1L	34	28
RAS/RAF	88	mTor/PI3K	60	CTLA4	33	27
RAS/RAF	88	mTor/PI3K	60	CDK4,6	32	26
RAS/RAF	88	mTor/PI3K	60	AURKA	29	24
RAS/RAF	88	mTor/PI3K	60	DNARepair	28	23
RAS/RAF	88	mTor/PI3K	60	ANGIO	27	22
RAS/RAF	88	mTor/PI3K	60	MET	27	22
RAS/RAF	88	mTor/PI3K	60	FGF	26	21
RAS/RAF	88	MET	40	CTLA4	32	26
RAS/RAF	88	CDK4,6	40	CTLA4	27	22
CDK4,6	63	RAS/RAF	51	ANGIO	24	20
CDK4,6	60	mTor/PI3K	48	AURKA	32	26
CDK4,6	60	mTor/PI3K	48	DNARepair	32	26
CDK4,6	60	mTor/PI3K	48	CTLA4	29	24
CDK4,6	60	mTor/PI3K	48	PARP	26	21
MEK	54	RAS/RAF	42	CTLA4	29	24
MEK	54	RAS/RAF	42	PD1L	28	23
MEK	54	RAS/RAF	42	mTor/PI3K	28	23
***RAS/RAF***	***88***	***mTor/PI3K***	***60***	***PD1L***	***34***	***28***
***ANGIO***	***56***	***RAS/RAF***	***41***	***PD1L***	***24***	***20***
***PD1L***	***63***	***mTor/PI3K***	***42***	***DNARepair***	***23***	***19***
***RAS/RAF***	***88***	***MET***	***40***	***PD1L***	***22***	***18***
***PD1L***	***63***	***mTor/PI3K***	***42***	***CDK4,6***	***21***	***17***
***PD1L***	***63***	***mTor/PI3K***	***42***	***ANGIO***	***21***	***17***
PD1L	63	mTor/PI3K	42	AURKA	20	16
PD1L	63	mTor/PI3K	42	IGF	19	15
PD1L	63	mTor/PI3K	42	FGF	18	15
PD1L	63	mTor/PI3K	42	MET	16	13

The frequency of activated interventional points in patients with activated immune-modulator targets is quite similar (Ras/Raf>Mek>Angiogenesis), whilst patients without their activation display a different profile: Her> mTor>, PARP> Polo-Aurora kinase.

Based on the magnitude and frequency of activation of interventional points and trends of co-activation, we indentified all possible triple combinations of targeted therapies, available for selection for an individualized therapeutic decision (Table 3 and Supplemental Table 8).

### Selection of triplet combinations

Focusing on the PDL1-activated group of patients, we determined the most frequent possible combinations. The six triplets that encompassed the greatest proportion of patients (>15% NSCLC) are described in Table 3.

The most frequent combinations that include PDL1 are activation of RAS/RAF, mTOR/PI3K and PDL1 (28% of all NSCLC) (Table 3). The other combinations are presented in the Discussion and as shown in Supplemental Tables 5 and 6, these combinations are overlapping in different patients, meaning that a given patient could potentially benefit from two or even more combinations. Overall, the six most frequent combinations that include PDL1 cover 63 out of 121 NSCLC patients, with no specific relationship to adenocarcinoma or squamous cell cancer histological types.

Combinations for patients with EGFR mutations or ALK rearrangements were less common because only a small proportion of our NSCLC displayed such aberrations.

Of interest, all the most commonly aberrantly activated interventional nodes can be targeted by drugs currently available in clinical trials or approved (as described in Table 1).

## DISCUSSION

We describe here a novel, simplified intervention mapping system (SIMS) method to efficiently identify the key activated pathways in a given cancer. The aim is to provide treatment guidance in the clinic in the form of a combination of three agents directed against the three intervention points most critical to the individual neoplasm.

In NSCLC, as well as in many cancer types, molecular characterization of the tumors has resulted in segmentation of nosological classifications, based previously on the organ of origin and histology, into a variety of molecular subtypes, often characterized by one specific driver molecular genetic alteration [3-14]. These driver alterations have successfully guided the development of novel targeted therapies for subsets of patients with NSCLC and *EGFR* mutations, as well as *ALK* or *ROS* translocations, and now a variety of other small subsets (*BRAF* mutations, *HER2* mutations, etc). However, this strategy has several limitations: *(i)* only a portion of tumors have an identified driver mutation, and many of these simple models may have been already described [15, 20]; *(ii)* there is no recognized strategy to efficiently pinpoint unrecognized drivers within the complex and multiple genomic alterations observed in most tumors; *(iii)* targeted treatments are not uniformly efficient even in these selected subgroups; *(iv)* the majority of tumors are actually driven by multiple aberrant genes [18, 19, 20], making the monotherapy paradigm unsuitable for most metastatic cancers; and *(v)* resistance uniformly emerges in a Darwinian manner in patients treated with a single targeted therapy.

The work presented here proposes a novel strategy to overcome these major limitations for the development of targeted therapies in patients with cancer. Using a dataset of 121 patients with NSCLC patients, and combining mutation information, CNV, and miRNA and mRNA expression in matched tumor and normal tissues, 24 intervention points potentially actionable by currently available targeted agents were identified, and allocated a score for each individual patient. We identified specific interventional points/nodes for drugs based on the pathways upregulated in each patient's cancer. This approach for prioritizing intervention points for a patient is simple. The basic premise is that, when the genes associated with an intervention point are more disturbed (in terms of sequence and/or expression level), the intervention point is more likely to be crucial to the tumor. From this, it follows that the more disturbed the genes of an intervention point are, the more likely it is that therapeutics targeting that point will benefit the patient. Accordingly, we have developed a family of simple scores that combine the level of gene expression in the tumor (relative to matched normal control), the mutations found in the intervention points' genes, CNVs and miRNAs expression levels. Intervention points with high scores varied across patients, but groups of tumors with similar combinations of high scores of activation points were identified, leading to a new dimensional classification of these tumors with potential predictive value for treatment efficacy. The technology described to delineate this score requires samples of tumor tissue and corresponding normal tissue, and molecular characterization tools that are readily available in many modern translational research facilities dedicated to cancer research. Its implementation in the clinical realm, integrating as well the rapid continuous improvements in technology, should therefore be readily feasible for the clinical trial setting.

The aim of this tool is to guide treatment decisions. We propose a new therapeutic approach of triple regimen therapies aimed at simultaneously blocking different biologic pathways and reducing the chance of developing secondary resistance. This simplified interventional mapping system (SIMS) identifies within the hallmarks of cancer [20] only signaling and regulatory pathways that can be targeted with available therapeutic agents. The principle of simplification is based on the activating signal that can be blocked by a class of drugs.

We therefore propose that combinations of three different classes of targeted agents acting on three independent intervention nodes will be necessary to derive a significant survival advantage for a given patient. One of these agents should ideally target the immune checkpoints, such as PDL1/PD1, or CTLA-4. The selection of a combination of three drugs derives from several considerations: *(i)* combinations of two drugs have shown modest improvements in survival compared to monotherapy; *(ii)* combinations of four or more agents will likely be excessively toxic [21]; and *(iii)* large enough subgroups of patients with the same three intervention points with high scores were identified in this work.

Focusing on the PDL1-activated group of patients, we determined the most frequent possible combinations (Table 5)]. Interest in PDL1-targeting agents is due to toxicity that differs from that of targeted agents, with less chance of overlap and hence amplified side effects when given together with two targeted agents such as tyrosine kinase inhibitors [21]. By far the most frequent combination involves activation of Ras/Raf, mTor/PI3K and PD1L, accounting for 28% of all NSCLCs. This major finding is consistent with the ATLAS report [12]: showing that recurrent aberrations in multiple key pathways and processes characterize lung adenocarcinoma. Among these were RTK/RAS/RAF pathway activation (76% of cases), PI(3)K-mTOR pathway activation (25%), p53 pathway alteration (63%), and alteration of cell cycle regulators (64%). Our data, obtained in an independent cohort, (representative for all NSCLC and not only adenocarcinoma), shows very similar results : Ras/Raf activated in 73% and CDK4,6 activated in 51% (Table 3). However, while previous reports affirm molecular segmentation of lung cancers, our data presents, for the first time, trends of coactivations in each individual patient, enabling definition and selection of combinations of therapies.

In addition to RAS/RAF, mTOR/PI3K and PDL1, other frequently co-activated pathways involving PDL1 are as follows [Supplemental Table 8]: *(i)* angiogenesis, RAS/RAF and PDL1 (20% NSCLC); *(ii)* PDL1, mTOR/PI3K and DNA Repair (19%); *(iii)* Ras/Raf, Met and PDL1 (18%); *(iv)* PDL1, mTor/PI3K and CDK4,6 (17%) and *(v)* PDL1, mTOR/PI3K, angiogenesis (17%). Overall, these six combinations cover 63 out of 121 patients with NSCLC regardless of histology. These combinations are overlapping in many patients, implying that each individual patient could potentially benefit from two or more combinations. Nevertheless, when taking into account all the possible combinations described in Table 3, it is worthwhile to mention that each patient may benefit from a therapeutic solution based on targeted agents available today. The finding is related to the new way of defining activated interventional nodes.

Integration of a variety omic datasets is advocated by many recently published opinions [13, 22, 23]. Consistent with the necessity of an integrative view, we used the power of multiple omic investigations in a novel way, defining a methodology and tools of integration focused on selection of combined therapies, which meets an urgent clinical need. This method aims to advance the paradigm of investigating individualized therapeutic options, in order to improve clinical outcome of patients with metastatic NSCLC.

Our system suggests that the combinations of three targeted treatments aimed at three intervention points with high scores enables selection of subgroups of patients of sufficient size so that clinical trials may be practically feasible. Although the analogy remains limited, it is noteworthy that tri-therapies with different modes of actions have previously been demonstrated to efficiently provide long-term control and/or cure for viral (HIV) and bacterial (TB) infections, Childhood leukemias also required combination therapy for long-term remission and cure. None of these ailments were cured with single agent treatment.

It should also be noted that our method for selecting triple-agent customized therapy for a patient assumes that impacting the three most “disturbed “ intervention points in a tumor would induce the best response. However, the SIMS framework can be used to test alternative hypotheses as well. For instance, one such alternative hypothesis is that simultaneous vertical targeting of a critical pathway by distinct types of drugs (such as antibodies and small molecule kinase inhibitors etc.) would more effectively extinguish the pathway.

One of the key forthcoming steps will be the implementation of clinical trials with innovative phase I/II telescoped designs in order to validate this strategy, and the delineation of the recommended doses of the combined targeted treatments. It will be particularly important to establish whether the treatments should be given simultaneously or sequentially, in order to both maximize antitumor effect and optimize tolerance.

Our work suggests a feasible prospective clinical trial that would benefit from several major assets. First, we identified at least six possible combinations of two targeted therapies together with an immune-modulator, applicable to at least half of NSCLC patients. Second, interrogation of dual biopsies of tumor and its normal counterpart, combined with a comprehensive systems biology investigation and innovative bioinformatics and scoring systems, may enable matching each individual patient with the most appropriate combination. Moreover, each patient will potentially benefit from different combinations, conferring a high chance of impacting survival of metastatic NSCLC.

One of the cornerstones of this new methodology is use of dual matched tumor and normal biopsies from the same patient, enabling the subtraction of transcriptomic background noise in each patient [13, 23]. Dual matched biopsies were implemented, for the first time in a clinical setting, in the WINTHER trial (http://clinicaltrials.gov/show/NCT01856296) of the Worldwide Innovative Networking (WIN) consortium (www.winconsortium.org) for personalized cancer therapy, and have proved feasible.

Nevertheless, this work does have several limitations. The number of patients with NSCLC was relatively small, and the data was collected retrospectively. Some important targets, such as EGFR and ALK, were not highlighted in the current results, mainly because they affected small numbers of our patients, and the effort here was to impact larger subgroups of individuals with NSCLC who may not benefit from EGFR and ALK inhibitors. It is probable that other populations, such as Asians, might have distinct patterns of aberrations, e.g., higher proportions with EGFR mutations. Fortunately, the algorithm permits adaptations to various populations and even to individuals. Many of the patients in our dataset had early-stage disease raising the question of extrapolation of the observations to late-stage patients. However, interestingly, our patients' tumors still expressed multiple perturbed interventional nodes, consistent with the concept that, by the time lung cancer is diagnosed, it already exhibits significant molecular heterogeneity. Additionally, we know of no other database of lung tumor and matched normal tissue from the same patient being comprehensively evaluated for mutations, gene expression, miRNA expression and CNV, enabling a comparison with the CHEMORES unique retrospective collection [13, 14]. In comparison, when analysing ATLAS sources [12], while the collection of 236 adenocarcinomas included matched tumor and normal tissues, only sequencing was performed on tumor and normal DNA; gene expression was done in a classic fashion, with microarrays investigating only the tumor RNA. However the frequencies of occurrence of major interventional points such as Ras/Raf, mTor/PI3K, and cell cycle regulators appear to be similar to that in our study.

Another limitation of our work is the challenge it presents for pre-clinical validation. Validation of the SIMS tool requires matched tumor and normal tissues from the same individual. Current preclinical models (cell lines in two or three dimensional culture, and xenografts in nude mice) cannot address this concept. Moreover, it is increasingly unclear as to the extent that these cell line or animal models can predict behavior *in vivo*, especially with immunomodulators that require an intact immune system, perhaps partially explaining the high attrition rate of drugs in development. Another limitation of the study relates to the targeted genomic sequencing that was performed. With the rapid evolution in technologies, more complete genomic sequencing should be applied to the next version of this analysis. Furthermore, next generations of the algorithm may also recognize distinctions between anomalies even within the same gene. For instance, not all p53 mutations behave as loss of function. Further, our understanding of the clinically relevant cut offs for expression of gene products such as PDL1 is still evolving, and correlating transcript expression to protein levels is in a nascent phase. Importantly, therefore, prospective validation of the algorithm and patterns of pathway abnormalities will be crucial. Furthermore, with the startling pace of advances in molecular methodological capabilities, algorithmical approaches to molecular complexity may need to be viewed as an iterative process, with prototypes being built and tested, and learning through the life cycle of the validation procedures.

In conclusion, this simplified intervention mapping system potentially reduces the enormous complexity of biological signals and pathway cross talk by devising a streamlined map that focuses only on the genes that are most indicative of drug target status, defined as “intervention points”. These intervention points consist of drug targets or groups of drug targets and some genes upstream of the drug targets that together reflect a specific biological activity that is actionable through currently available therapeutic interventions. This simplified mapping and scoring tool provides a new way of integrating genomic data, not previously described, even in state-of-the art reports [22] SIMS converts thousands of genomic measurements into a simple format, that can potentially be exploited by clinicians, and may facilitate rationally based selection of targeted agents for the treatment of individual patients and, most importantly, selection of triple therapy combinations.

To summarize, targeted monotherapies lead systematically to resistance. To overcome resistance, we present a novel therapeutic interventional mapping system and algorithm, based on integration of genomic and transcriptomic data that may allow deployment of customized combinations of therapy. Therefore, the integrative omic approach together with the SIMS algorithmic strategy and tools presented herein can be realistically exploited to inform the development of next generation clinical trials addressing personalized combinations of targeted cancer treatments, a strategy with the objective of impacting survival and of progress towards a curative approach for patients with metastatic NSCLC. To this aim, the WIN Consortium has aligned numerous stakeholders including academia, the pharmaceutical industry, biotechnology, and health payors in order to prepare and launch the SPRING (Survival Prolongation by Rationale Innovative Genomics) lung cancer clinical trial, whose objective will be to prospectively validate the SIMS concept. Importantly, the same strategy may also be applicable to other deadly malignancies.

## MATERIALS AND METHODS

### Patients and tissue samples

The present study used *in silico* data generated and published by the CHEMORES initiative (www.chemores.org), which is an EU funded (FP6) Integrated Project. Tissue samples from a cohort of 121 patients who underwent complete surgical resection at the Institut Mutualiste Montsouris (Paris, France) between 30 January 2002 and 26 June 2006 were analyzed.

### Characteristics of NSCLC cohort

The median age of patients was 63 years (range, 41-85 years); 89 patients (73%) were men (Supplemental Table 1). The histopathology of all tumors was reviewed by the same pathologist. The most common subtypes of tumor were adenocarcinoma and squamous cell cancer. Using the new 7th edition TNM staging, 56 were stage I, 24 stage II, 27 stage III and 4 stage IV. Adjuvant platinum based chemotherapy was administered to 61 patients. Fifty-nine patients experienced a relapse. Two-year relapse-free survival was 64%, and the median time to recurrence for the cohort was 5.2 years. After a median follow up of 40 months (range, 0-92 years) 36 patients had died and 23 patients were alive with recurrence.

This study was performed using snap-frozen tumor and matched normal lung tissue, from the same patients, after curative surgery. Samples were handled according to the Tumor Analysis Best Practices Working Group [14]. Haematoxylin and eosin stained frozen sections, taken before and after the cutting of slides for molecular analysis, revealed a median cell content of 85% (an inter-quartile range of 65% to 95%). All tissues were banked after written informed patient consent, and the study was approved by the Ethics Committee of Gustave Roussy (GR). A full description of the genomic investigation is available at Lazar et al. [13], and in Supplemental Methods [15-17] and data. [Supplemental Tables 2, 3, and 4 describe the genes, mutations, and miRNAs analyzed]. The microarray data related to this study have been submitted to the Array Express data repository at the European Bioinformatics Institute (http://www.ebi.ac.uk/arrayexpress/) under the accession numbers E-MTAB-1132 (GE), E-MTAB-1133 (CGH) and E-MTAB-1134 (MIR).

### Scoring/ranking of activated interventional points

(Figure 2). A score of 1 to 10 is assigned. If a mutation is present, a score of 10 is given and the transcriptomic information is not used. If there is no mutation, the CNV and transcriptomic information (mRNA adjusted by miRNA) are scored.

### The algorithm

The mathematical modelling and scoring system aims to give a score (1 to 10) based on integration of omics data, sequencing, gene expression, miRNA and copy number variations determined as differences between tumor and normal tissues, individually for each patient. Scoring enables identification and ranking of activated pathways, and the overall concept is that such activated pathways should be blocked with combined targeted therapies.

### The mathematical model

The initial model was established on the basis of a retrospective dataset from 121 patients with NSCLC for whom sequencing, CNVs, and tumor vs. normal gene expression were available. Using these data, an algorithm that provides a score of activation for each of the simplified pathways for the patient and factors in all of the above-mentioned structural and functional results has been established. The principle of the algorithm is described below:

### Scoring

Scoring is performed by using an intuitive algorithm that integrates four types of molecular investigations of tumor and normal tissues (genomics, mRNA expression, miRNA and copy number variations).

### Mutations

In version 1, we used a very limited set of sequencing data, including only the genes/mutations used currently in clinical care of *NSCLC: EGFR, KRAS, BRAF, PI3KCA*, and *HER2*. Additionally we sequenced p53, the most frequently mutated gene in lung (and most other solid) tumors. When a mutation is detected, the algorithm assigns the maximal score 10 in the corresponding simplified pathway.

### Gene expression

For each interventional node, mRNA steady state level in tumor *vs*. normal is used to calculate a mean fold change of the pathway from the values of individual fold change (Fc) of tumor vs normal for each gene of the interventional nodes. For calculating the mean/average fold change of intervention point *k,* denoted as *E_k_*, the fold changes of differentially expressed genes with a fold change of at least 1.3 are used. Based on Agilent microarrays specifications, the threshold of 1.3 was considered as the lowest conferring accurate detection, since all Fc values were obtained by combining two dyes swap microarray experiments. In other words, for each intervention point, an average fold-change of the genes *i* of the intervention point *k* is calculated, trimming values with a threshold of <1.3. Formally, we calculate *E_k_* as the following: let *M_k_* denote the set of genes that belong to intervention point *k*, and *m_k_* denote the subset of *M_k_* that includes only differential expressed genes with an absolute fold change >1.3. *E_k_* is the average of the fold change of the genes *m_k_*. *m_k_* = {*i*|*i* ∈ *M_k_ and* |*F_i_*| > 1.3}. We then calculate the mean expression level for all the genes in *m_k_*:

$$E_{k}=\bar{F_{i}}$$

wherein *i* ∈ *m_k_*. In other words, the fold change for a particular intervention point is the average or arithmetic mean of the fold changes of genes belonging to the intervention point as defined in Table 1 and having a fold change T vs N of 1.3 or more. In particular, in order to compare the fold changes of different intervention points, a relative scoring, e.g., from 1 to 10, is generated based on the decile calculation using as calibrator the data obtained from all 121 NSCLC patients. Values of individual mean fold changes for each simplified pathway are ranked in the retrospective set of data of 121 patients with NSCLC, used as a calibrator. As shown in Figure 2B the range of changes is different from one pathway to another. In order to compare them, we generated a relative scoring from 1 to 10 based on the decile calculation.

### Combining mRNA and miRNA measurements

To adjust for possible miRNA intervention in translation, we penalized discordance between miRNA and its target mRNA. For each of the genes of Table 1 (and Supplemental Table 2) that belong to the intervention points or a set thereof, we determined the miRNAs most likely to be involved in their regulation using Target scan {http://www.targetscan.org/}, selecting the top 5 miRNAs for each gene. Supplemental Table 4 provides a list of the top 5 miRNAs for the genes of Table 1. For each gene *i*, a mean miRNA fold-change can be calculated, which is denoted *A_i_*, by averaging the fold changes of the 5 miRNAs (or less if less than 5 miRNAs are identified) that are most likely to target gene *i.* Then, for each gene, a mean miRNA T*v*N fold change is determined. Then, a corrected fold change of a gene of an intervention point is calculated by dividing the mRNA fold change of tumor versus normal of the gene (mRNA T*v*N fold change) by the mean fold change for the miRNAs of the gene (mean miRNA T*v*N fold change). The corrected fold change of a gene is then used to calculate the fold change for a particular pathway by using it in the calculation of the average fold changes of the genes belonging to the pathway as defined in Table 1 and having a fold change T vs N of 1.3 or more. Based on the corrected fold change of pathways, a corrected score, e.g., a score 1 to 10 is generated based on deciles.

### Copy number variation

When amplification is detected, we multiply the value of the mRNA expression fold change for each gene by the value of the fold change in copy number. Then we generate the corrected mean fold change of pathways and the deciles score. However CNV had little impact in our analysis of the 183 genes in 121 patients.

## SUPPLEMENTARY MATERIALS TABLES


