# Supplementary material for: A simplified interventional mapping system (SIMS) for the selection of combinations of targeted treatments in non-small cell lung cancer
Source: Oncotarget. 2015 Apr 3;6(16):14139–52. doi: 10.18632/oncotarget.3741 (PMC4546456; doi:10.18632/oncotarget.3741)
Supplement: Supplementary file 1 [file oncotarget-06-14139-s001.pdf]

A simplified interventional mapping system (SIMS) for the selection of combinations of targeted treatments in non-small cell lung cancer

Supplementary Material

SUPPLEMENTAL DATA

Methodology of genomic investigation:

Oligonucleotide aCGH

DNA samples were extracted from tissues using Qiagen QIAamp DNA Mini kit (Qiagen, Hilden, Germany). In each case, the normal tissue sample was used as the reference to its corresponding tumor sample. DNA was restriction digested and controlled by Agilent Bioanalyzer on DNA 7500 chips (Agilent Technologies, Santa Clara, CA, USA). The fragmented reference and test DNA were labelled with Cy3-dUTP or Cy5-dUTP, respectively, using Agilent Genomic DNA Labelling Kit PLUS. Samples were purified using Microcon YM-30 filters (Millipore, Billerica, MA). Hybridization was carried out on Agilent 244K arrays for 24 hours at 65℃ in a rotating oven (Robbins Scientific, Mountain View, CA) at 20rpm, followed by appropriate washing steps. Scanning was performed with an Agilent G2505C DNA Microarray scanner using default parameters. Quantification of Cy5 and Cy3 signals from scans was performed with Feature Extraction v10.5.1.1 (Agilent Technologies) using default parameters.

aCGH data processing and analysis

Resulting raw signals and log2 (ratio) profiles were normalized and centered according to their dye composition (Cy5/Cy3) and local GC content. These profiles were segmented with the Circular Binary Segmentation algorithm (16) through its implementation in the DNAcopy package for R v2.8.1 using default parameters. DNA copy number imbalances were detected considering a minimum of 3 consecutive probes and a minimal absolute amplitude threshold that was specific for each profile, accordingly with its internal noise. This specific internal noise was computed as one-fourth of the median of the absolute log2 (ratio) distances across consecutive probes on the genome. Of the 121 aCGH hybridizations performed, 17 were discarded: 7 due to their clinical annotations, 2 due to anomalies in their normal reference, and 8 due to the bad quality of their profile, resulting in 111 usable profiles. All aCGH coordinates in this study are mapped against the human genome as defined by the UCSC build hg18.

To assess the discovery of the genomic regions with differential anomalies between the adenocarcinoma, large cell cancer and squamous cell cancer populations,

ANOVA tests were performed on the segmented aCGH dataset. To account for multiple testing, p-values were transformed to false discovery rate (FDR) (17)

Gene expression and microRNA microarray assay

The lysis of 40 to 50 frozen sections of 10 micron-thickness, cut from each NSCLC tissue sample was done using a Polytron homogenizer (Ultraturrax, IMLAB, Lille, France). The RNA extraction was performed with TRIzol® Reagent protocol (Invitrogen, Carlsbad, CA, USA). Total RNA was quantified and qualified with Nanodrop ND-1000 spectrometer and Bioanalyzer-2100 (Agilent Technologies).

For dual color Cy3 (normal samples) and Cy5 (tumor samples) labelling, Agilent Fluorescent Low Input Linear Amplification kit adapted for small amounts of total RNA (500 ng total RNA per reaction) was used, followed by purification of labelled probes by Qiagen RNeasy Mini kit and by a protocol provided by Agilent. Gene expression profiling was performed with dye-swap, using dual-color 244K Human exon array from Agilent (custom design with the content of the 44K Human genome plus 195000 probes, one for each exon as defined in refGene list of UCSC build hg18 (http://genome.ucsc.edu/)). Hybridization was carried out for 17 hours at 65℃ at 10 rpm, followed by washing steps. Scanned microarray images were analyzed by using Feature Extraction software version 10.5.1.1 (Agilent).

For the microRNA analysis, normal and tumor samples were hybridized on separate arrays. Agilent miRNA Microarray System with miRNA complete labelling and hybridization kit was used for Cy3 labelling. Briefly, isolated total RNAs were dephosphorylated, labelled with pCp-Cy3 and hybridized to Agilent 8x15K arrays for 20h at 55℃ in a rotating oven (Robbins Scientific) at 20 rpm. Slides were washed and scanned for gene expression using an Agilent G2565C DNA microarray scanner using defaults parameters.

Gene mutations analysis

Sequencing was performed at Gustave Roussy and at the Royal Institute of Technology (Stockholm, Sweden). DNA was extracted with QIAamp DNA Mini Kit (Qiagen, Hilden, Germany). After PCR amplification of target exons, sequencing reactions were carried out using the BigDye® Terminator Cycle Sequencing Kit (Applied Biosystems, Forster City, CA). The primer sequences are available on

request. Sequencing reactions were run on a 48-capillary 3730 DNA Analyzer®. Sequence analysis and alignment was performed with SeqScape® software (Applied Biosystems). All detected mutations were confirmed in at least one independent PCR reaction. In all 121 samples, full coding sequences of exons including oncogenic mutational hotspots were analyzed corresponding to: TP53 (NM_000546.4) exons 5-8; KRAS (NM_004448.2) exons 2 and 3; EGFR (NM_005228.3) exons 18-21; PIK3CA (NM_006218.2) exons 10 and 21; BRAF (NM_004333.4) exon 15; ERBB2 (NM_004448.2) exons 18, 20-24; KDR (NM_002253.1) exons 2, 26, 27 and 30; and AKT1 (NM_005163.2) exon 4.

Gene-expression data processing and normalisation

All processing methods used for gene expression analysis were performed on the median signal from Agilent Feature Extraction raw data files using functions and packages collected in the R Bioconductor project (Gentleman et al. *Genome Biology*, 5: R80) as well as custom written routines.

For gene expression data, dye-swap arrays were first combined (by taking the average of intensities) to obtained only one array per condition. This combination has the result of centering the M values (log2ratios) on zero. Then, flagged spots as well as control spot were removed. Normalization was then performed using the *normalizeWithinArrays* function from R package LIMMA (18).

For miRNA data, control spots were systematically removed, and flagged spots (gIsFeatNonUnifOL and gIsSaturated columns from raw files) were considered as missing values (“NA”). Array normalization was performed using the least-variant-set method (Suo et al. *RNA* 2010 Dec; 16(12): 2293-303).

Differential expression analyses of miRNA expression

To assess differentially-expressed miRNA, we first estimated the fold changes and standard errors between two groups of samples by fitting a linear model for each probe with the lmFit function of LIMMA package in R. Then they applied an empirical Bayes smoothing to the standard errors from the linear model previously computed with eBayes function.

SUPPLEMENTAL TABLES

Supplemental Table 1 *- Characteristics of the patients with NSCLC in the study population*

	N=121 (100%)
Age median (range) (years)	63 (40.9-84.6)
Males n (%)	89 (72%)
Smoking	
Current	63 (52%)
Former	51 (42%)
Never	7 (6%)
Histology	
Adenocarcinoma	56 (46%)
Squamous cell lung cancer	50 (42%)
Large cell cancer	12 (10%)
Other	3 (2%)
Stage	
1	56 (46%)
2	24 (19%)
3	27 (22%)
4	4 (3%)
Adjuvant Chemo (%)	61 (50%)

Supplemental Table 2: *Detailed List of genes of interventional points of SIMS*

Pathway	Symbol	GeneID	Name	Refseq
HER	EGF	1950	epidermal growth factor	NM_001963
	TGFA	7039	transforming growth factor, alpha	NM_003236
	AREG	374	amphiregulin	NM_001657
	EREG	2069	epiregulin	NM_001432
	HBEGF	1839	heparin-binding EGF-like growth factor	NM_001945
	BTC	685	betacellulin	NM_001729
	NRG1	3084	neuregulin 1	AF176921; NM_004495
	NRG2	9542	neuregulin 2	ENST00000544729; NM_013982
	NRG4	145957	neuregulin 4	NM_138573
	EGFR	1956	epidermal growth factor receptor	NM_201283; NM_201282; NM_005228
	ERBB2	2064	v-erb-b2 avian erythroblastic leukemia viral oncogene homolog 2	NM_001005862; AB025286
	ERBB3	2065	v-erb-b2 avian erythroblastic leukemia viral oncogene homolog 3	NM_001982; NM_001005915
	ERBB4	2066	v-erb-b2 avian erythroblastic leukemia viral oncogene homolog 4	NM_005235
CDK4,6	CDK4	1019	cyclin-dependent kinase 4	NM_000075
	CDK6	1021	cyclin-dependent kinase 6	NM_001259
	CCND1	595	cyclin D1	NM_053056
	CCND2	894	cyclin D2	NM_001759
	CCND3	896	cyclin D3	NM_001760
	CDKN2A,	1029	cyclin-dependent kinase inhibitor 2A	NM_058197; NM_000077
	CDKN2B	1030	cyclin-dependent kinase inhibitor 2B	NM_004936
	CCNE1	898	cyclin E1	NM_001238
	CCNE2	9134	cyclin E2	NM_057749
	RB1	5925	retinoblastoma 1	NM_000321
PLK / AURK / Kinesins	PLK1	5347	polo-like kinase 1	NM_005030
	AURKA	6790	aurora kinase A	NM_198433
	BORA	79866	bora, aurora kinase A activator	NM_024808
	ILK	3611	integrin-linked kinase	NM_001014795
	KIF11	3832	kinesin family member 11	NM_004523
ANGIOGENESIS	VEGFA	7422	vascular endothelial growth factor A	NM_001025370; NM_001025366
	VEGFB	7423	vascular endothelial growth factor B	NM_003377
	VEGFC	7424	vascular endothelial growth factor C	NM_005429
	VEGFD	2277	c-fos induced growth factor (vascular endothelial growth factor D)	NM_004469
	FLT1	2321	fms-related tyrosine kinase 1	NM_001160031; NM_002019
	KDR	3791	kinase insert domain receptor (a type III receptor tyrosine kinase)	NM_002253
	FLT4	2324	fms-related tyrosine kinase 4	ENST00000376868; NM_002020

	PDGFA	5154	platelet-derived growth factor alpha polypeptide	NM_002607; NM_033023
	PDGFB	5155	platelet-derived growth factor beta polypeptide	NM_002608
	PDGFRA	5156	platelet-derived growth factor receptor, alpha polypeptide	NM_006206
	PDGFRB	5159	platelet-derived growth factor receptor, beta polypeptide	NM_002609
	Kit	3815	v-kit Hardy-Zuckerman 4 feline sarcoma viral oncogene homolog	NM_000222; NM_001093772
ANGIOPOIETINS	THBS1	7057	thrombospondin 1	NM_003246
	TGFB1	7040	transforming growth factor, beta 1	NM_000660
	ANGPT1	284	angiopoietin 1	NM_001146
	ANGPT2	285	angiopoietin 2	NM_001147
	ANGPTL1	9068	angiopoietin-like 1	NM_004673
	ANGPT4	51378	angiopoietin 4	NM_015985
	TIE1	7075	tyrosine kinase with immunoglobulin-like and EGF-like domains 1	NM_005424
	TEK	7010	TEK tyrosine kinase, endothelial	NM_000459
IMMUNO-Modulator	CD274 or PD1L	29126	CD274 molecule<br>programmed cell death ligand 1	NM_014143
	PDCD1LG2	80380	programmed cell death 1 ligand 2	NM_025239
	PDCD1	5133	programmed cell death 1	NM_005018
	CTLA4	1493	cytotoxic T-lymphocyte-associated protein 4	NM_005214
	LAG3	3902	lymphocyte-activation gene 3	NM_002286
PI3K	PIK3CA	5290	phosphatidylinositol-4,5-bisphosphate 3-kinase, catalytic subunit alpha	NM_006218
	PIK3CB	5291	phosphatidylinositol-4,5-bisphosphate 3-kinase, catalytic subunit beta	NM_006219
	PIK3CD	5293	phosphatidylinositol-4,5-bisphosphate 3-kinase, catalytic, catalytic subunit delta	NM_005026
	PIK3CG	5294	phosphatidylinositol-4,5-bisphosphate 3-kinase, catalytic subunit gamma	NM_002649
	PIK3C2B	5287	phosphatidylinositol-4,5-bisphosphate 3-kinase, catalytic subunit type 2 beta	NM_002646; ENST00000367184
	PRKCB	5579	protein kinase C, beta	NM_002738
	PRKCA	5578	protein kinase C, alpha	NM_002737
	PIK3R1	5295	phosphoinositide-3-kinase, regulatory subunit 1 (alpha)	NM_181523
	PIK3R2	5296	phosphoinositide-3-kinase, regulatory subunit 2 (beta)	NM_005027
	PIK3R3	8503	phosphoinositide-3-kinase, regulatory subunit 3 (gamma)	NM_003629
MET	HGF	3082	hepatocyte growth factor (hepapoietin A; scatter factor)	NM_001010934; NM_001010931
	MET	4233	met proto-oncogene	NM_000245
	AXL	558	AXL receptor tyrosine kinase	NM_021913
	MST1R	4486	macrophage stimulating 1 receptor (c-met-related tyrosine kinase)	NM_002447
MEK	MAP2K1	5604	mitogen-activated protein kinase kinase 1, E3 ubiquitin protein ligase	NM_002755
	MAP2K2	5605	mitogen-activated protein kinase kinase 2	NM_030662
	MAP2K3	5606	mitogen-activated protein kinase kinase 3	NM_145109; ENST00000534743
	MAP2K4	6416	mitogen-activated protein kinase kinase 4	NM_003010
	MAP3K1	4214	mitogen-activated protein kinase kinase kinase 1	NM_005921
	MAP3K2	10746	mitogen-activated protein kinase kinase kinase 2	NM_006609
	MAP3K3	4215	mitogen-activated protein kinase kinase kinase 3	NM_203351
	MAP3K4	4216	mitogen-activated protein kinase kinase kinase 4	NM_005922; NM_006724

ERK	MAPK3	5595	mitogen-activated protein kinase 3	NM_002746
	MAPK1	5594	mitogen-activated protein kinase 1	NM_138957
	KSR1	8844	kinase suppressor of ras 1	NM_014238
	MAPK11	5600	mitogen-activated protein kinase 11	NM_002751
ANTI-APOPTOSIS	BCL2	596	B-cell CLL/lymphoma 2	NM_000633; NM_000657
	BCL2L1	598	BCL2-like 1	NM_138578
	BIRC5	332	baculoviral IAP repeat containing 5	NM_001012271
	XIAP	331	X-linked inhibitor of apoptosis	NM_001167
	BAK1	578	BCL2-antagonist/killer 1	NM_001188
FGF	FGF1	2246	fibroblast growth factor 1 (acidic)	NM_000800; NR_026696
	FGF2	2247	fibroblast growth factor 2 (basic)	NM_002006
	FGF3	2248	fibroblast growth factor 3	NM_005247
	FGF4	2249	fibroblast growth factor 4	NM_002007
	FGF5	2250	fibroblast growth factor 5	NM_004464; NM_033143
	FGF6	2251	fibroblast growth factor 6	NM_020996
	FGF7	2252	fibroblast growth factor 7	NM_002009
	FGF8	2253	fibroblast growth factor 8 (androgen-induced)	NM_033163
	FGF9	2254	fibroblast growth factor 9	NM_002010
	FGF10	2255	fibroblast growth factor 10	NM_004465
	FGF11	2256	fibroblast growth factor 11	NM_004112
	FGF12	2257	fibroblast growth factor 12	NM_004113
	FGF13	2258	fibroblast growth factor 13	NM_004114
	FGF14	2259	fibroblast growth factor 14	NM_175929
	FGFR1	2260	fibroblast growth factor receptor 1	ENST00000496296; NM_023110; NM_001174066
	FGFR2	2263	fibroblast growth factor receptor 2	ENST00000359354; NM_022970
	FGFR3	2261	fibroblast growth factor receptor 3	NM_000142
	FGFR4	2264	fibroblast growth factor receptor 4	NM_213647

mTOR - AKT- PTEN-	mTor	2475	mechanistic target of rapamycin (serine/threonine kinase)	NM_004958
	AKT1	207	v-akt murine thymoma viral oncogene homolog 1	NM_005163
	AKT2	208	v-akt murine thymoma viral oncogene homolog 2	NM_001626
	PTEN	5728	phosphatase and tensin homolog	NM_000314
Modulators MTKPT	TSC1	7248	tuberous sclerosis 1	NM_000368; ENST00000403810
	TSC2	7249	tuberous sclerosis 2	NM_000548; NM_001077183
	STK11	6794	serine/threonine kinase 11	NM_000455
	PIM1	5292	pim-1 oncogene	NM_002648
	PIM2	11040	pim-2 oncogene	NM_006875
	PIM3	415116	pim-3 oncogene	NM_001001852
RAS	KRAS	3845	Kirsten rat sarcoma viral oncogene homolog	NM_033360; NM_004985
	NRAS	4893	neuroblastoma RAS viral (v-ras) oncogene homolog	NM_002524
	HRAS	3265	Harvey rat sarcoma viral oncogene homolog	NM_005343

RAF	RAF1	5894	v-raf-1 murine leukemia viral oncogene homolog 1	NM_002880
	BRAF	673	v-raf murine sarcoma viral oncogene homolog B	NM_004333
TELOMERASE	TERT	7015	telomerase reverse transcriptase	NM_198253
	TERC	7012	telomerase RNA component	NR_001566
	TEP1	7011	telomerase-associated protein 1	NM_007110
	HSP90AA1	3320	heat shock protein 90kDa alpha, class A member 1	NM_001017963; NM_005348
	DKC1	1736	dyskeratosis congenita 1, dyskerin	NM_001363
	PTGES3	10728	prostaglandin E synthase 3	NM_006601
IGF & Warburg	IGF1	3479	insulin-like growth factor 1 (somatomedin C)	NM_000618
	IGF2	3481	insulin-like growth factor 2 (somatomedin A)	NM_000612
	IGF1R	3480	insulin-like growth factor 1 receptor	NM_000875
	IGF2R	3482	insulin-like growth factor 2 receptor	NM_000876
	INSR	3643	insulin receptor	NM_000208
	IRS1	3667	insulin receptor substrate 1	NM_005544
	PKM	5315	pyruvate kinase, muscle	NM_001206796.1
WNT	CDH1	999	cadherin 1, type 1, E-cadherin (epithelial)	NM_004360
	CTNNA1	1495	catenin (cadherin-associated protein), alpha 1, 102 kDa	NM_001903
	CTNNB1	1499	catenin (cadherin-associated protein), beta 1, 88 kDa	NM_001904; NM_001098210
	WNT 1	7471	wingless-type MMTV integration site family, member 1	NM_005430
	FZD1	8321	frizzled class receptor 1	NM_003505
	WNT5A	7474	wingless-type MMTV integration site family, member 5A	NM_003392
	WNT5B	81029	wingless-type MMTV integration site family, member 5B	NM_030775
	FZD5	7855	frizzled class receptor 5	NM_003468
	WIF1	11197	WNT inhibitory factor 1	NM_007191
	DKK1	22943	dickkopf WNT signaling pathway inhibitor 1	NM_012242

PARP	PARP1	142	poly (ADP-ribose) polymerase 1	NM_001618; ENST00000366790
	BRCA1	672	breast cancer 1, early onset	NM_007300
	XRCC1	7515	X-ray repair complementing defective repair in Chinese hamster cells 1	NM_006297
	RAD54L	8438	RAD54-like (S. cerevisiae)	NM_003579
	RAD54B	25788	RAD54 homolog B (S. cerevisiae)	NM_012415; NM_001205262
	ATM	472	ataxia telangiectasia mutated	NM_000051; ENST00000389511
	ATR	545	ataxia telangiectasia and Rad3 related	NM_001184
	CHEK1	1111	checkpoint kinase 1	NM_001114121
	CHEK2	11200	checkpoint kinase 2	NM_145862; NM_001005735
	WEE1	7465	WEE1 G2 checkpoint kinase	NM_003390
HDAC	HDAC1	3065	histone deacetylase 1	NM_004964
	HDAC2	3066	histone deacetylase 2	NM_001527
	HDAC3	8841	histone deacetylase 3	NM_003883
	HDAC4	9759	histone deacetylase 4	NM_006037
	HDAC5	10014	histone deacetylase 5	NM_001015053
JAK-STAT	JAK1	3716	Janus kinase 1	NM_002227
	JAK2	3717	Janus kinase 2	NM_004972

	STAT1	6772	signal transducer and activator of transcription 1, 91 kDa	NM_139266
	STAT2	6773	signal transducer and activator of transcription 2, 113 kDa	NM_005419
	STAT3	6774	signal transducer and activator of transcription 3 (acute-phase response factor)	NM_213662
	SOCS1	8651	suppressor of cytokine signaling 1	NM_003745
HEDGEHOG	SHH	6469	sonic hedgehog	NM_000193
	PTCH1	5727	patched 1	NM_001083602; ENST00000375290
	SMO	6608	smoothened, frizzled class receptor	NM_005631
	STK36	27148	serine/threonine kinase 36	NM_015690
	PRKACA	5566	protein kinase, cAMP-dependent, catalytic, alpha	NM_002730
	SUFU	51684	suppressor of fused homolog (Drosophila)	NM_016169; NM_001178133
	GLI1	2735	GLI family zinc finger 1	NM_005269
DNA REPAIR	ERCC1	2067	excision repair cross-complementation group 1	NM_202001
	RAD52	5893	RAD52 homolog (S. cerevisiae)	NM_134424; ENST00000545967
	XRCC4	7518	X-ray repair complementing defective repair in Chinese hamster cells 4	NM_022550
	RAD51	5888	RAD51 recombinase	NM_002875
	BRCA1	672	breast cancer 1, early onset	NM_007300
	NEDD8	4738	neural precursor cell expressed, developmentally down-regulated 8	NM_006156
	NAE1	8883	NEDD8 activating enzyme E1 subunit 1	NM_001018159
NOTCH	NOTCH1	4851	notch 1	NM_017617
	Adam17	6868	ADAM metallopeptidase domain 17	NM_003183
	PSEN1	5663	presenilin 1	NM_000021; ENST00000394157
	NCSTN	23385	nicastrin	NM_015331
	JAG1	182	jagged 1	NM_000214
	SRRT	51593	serrate RNA effector molecule homolog (Arabidopsis)	NM_001128853; NM_015908; NM_001128854
	APH1A	51107	APH1A gamma secretase subunit	NM_016022; NM_001077628
Others	ROS1	6098	c-ros oncogene 1 , receptor tyrosine kinase	ENST00000403284; NM_002944
	ALK	238	anaplastic lymphoma receptor tyrosine kinase	NM_004304
	RET	5979	ret proto-oncogene	NM_020630; NM_020975
	UBA1	7317	ubiquitin-like modifier activating enzyme 1	NM_003334

Supplemental Table 3 : *List of gene mutations*

BRAF

Nucleotide	Protein	
c.1799 T>W	p.Val600Glu	V600E
c. 1798 G>R<br>c.1799 T>W	p.Val600Lys	V600K
c.1799 T>W c.1800G>R	p.Val600Glu	V600E
c.1780 G>R	p.Asp594Asn	D594N

EGFR

Nucleotide	Protein		Effect of first generation EGFR inhibitors
c.2156G>C	p.Gly719Ala	G719A	Sensitivity
c.2155 G>K	p.Gly719Cys	G719C	Sensitivity
c.2117 T>Y	p.Ile706Thr	I706T	Sensitivity
c.2125 G>R	p.Glu709Lys	E709K	Sensitivity
c.2126 A>M	p.Glu709Ala	E709A	Sensitivity
c.2174 C>Y	p.Thr725Met	T725M	Sensitivity
c.2165C>M	p.Ala722Glu	A722E	Sensitivity
c.2235_2249 del	p.Glu746_Ala750del	Deletion E746-A750	Sensitivity
c.2236_2250 del	p.Glu746_Ala750del	Deletion E746-A750	Sensitivity
c.2240_2254del	p.Leu747_Thr751del	Deletion L747-T751	Sensitivity
c.2240_2257 del	p.Leu747_Pro753delinsSer	Deletion L747-P753 Insertion S	Sensitivity
c.2237_2251del	p.Glu746_Thr751delinsAla	Deletion E746-T751 Insertion A	Sensitivity
c.2239_2248delinsC	p.Leu747_Ala750delinsPro	Deletion L747-A750 Insertion P	Sensitivity
c.2239_2251delinsC	p.Leu747_Thr751delinsPro	Deletion L747-T751 Insertion P	Sensitivity
c.2237_2255 delinsT	p.Glu746_Ser752delinsVal	Deletion E746-S752 Insertion V	Sensitivity
c.2214_2231dup	p.Ile740_Lys745dup	Duplication I740-K745	Sensitivity
c.2254_2277 del	p.Ser752_Ile759del	Deletion S752-I759	Sensitivity
c.2219_2236dup	p.Lys745_Glu746insValProValAlaIleLys	K745-E746 Insertion VPVAIK	Sensitivity
c.2277 C>S	p.Ile759Met	I759M	Sensitivity
c.2239_2256delinsCAA	p.Leu747_Ser752delinsGln	Deletion L747-S752 Insertion Q	Sensitivity
c.2369C>Y	p.Thr790Met	T790M	Resistance
c.2317_2318insACC	p.His773dup	Duplication H773	Resistance
c.2317_2318ins12	p.Pro772_His773insLeuGlyAsnPro	P772-H773 insertion LGNP	Resistance
c.2315_2326dup	p.Pro772_Cys775dup	Duplication P772-C775	Resistance
c.2300_2308 dup	p.Ala767_Val769dup	Duplication A767-V769	Resistance
c.2303_2311 dup	p.Ser768_Asp770dup	Duplication S768-D770	Resistance
c.2303_2311dup	p.Ser768_Asp770dup	Duplication S768-D770	Resistance
c.2335G>T	p.Gly779Cys	G779C	Resistance
c.2573 T>K	p.Leu858Arg	L858R	Sensitivity
c.2582 T>W	p.Leu861Gln	L861Q	Sensitivity

KRAS-NRAS

Nucleotide	Protein	
c.34 G>K	p.Gly12Cys	G12C
c.35 G>R	p.Gly12Asp	G12D
c.35 G>K	p.Gly12Val	G12V
c.35 G>S	p.Gly12Ala	G12A
c.34 G>R	p.Gly12Ser	G12S
c.34 G>S	p.Gly12Arg	G12R
c.38 G>R	p.Gly13Asp	G13D
c.37 G>K	p.Gly13Cys	G13C
c.182 A>W	p.Gln61Leu	Q61L
c.182 A>R	p.Gln61Arg	Q61R
c.183 A>M	p.Gln61His	Q61H
c.176 C>S	p.Ala59Gly	A59G

c.175 G>R	p.Ala59Thr	A59T
c.176 C>M	p.Ala59Glu	A59E

ERBB2

Nucleotide	Protein	
c.2313_2324dup	p.Tyr772_Ala775dup	Duplication Y772-A775
c.2318_2319insGATGGCATACGT	p.Tyr772_Ala775dup	Duplication Y772-A775
c.2326_2327insTGT	p.Gly776delinsValCys	Deletion G776<br>Insertion VC
c.2331_2339dup	p.Gly778_Pro780dup	Duplication G778-P780

PIK3CA

Nucleotide	Protein	
c.1624 G>R	p.Glu542Lys	E542K
c.1633G>R	p.Glu545Lys	E545K
c.3140A>R	p.His1047Arg	H1047R
c.3140A>W	p.His1047Leu	H1047L
c.2959 G>R	p.Ala987Thr	A987T
c.3052G>A	p.Asp1018Asn	D1018N
c.3080 C>Y	p.Ala1027Val	A1027V
c.3131A>R	p.Asn1044Ser	N1044S

Supplemental Table 4 : *List of miRNA*

Pathway	Symbol	GeneID	miRNAs
HER	EGF	1950	hsa-miR-4673; hsa-miR-485-5p; hsa-miR-647 ; hsa-miR-4742-5p ; hsa-miR-4797-5p
	TGFA	7039	hsa-miR-3147; hsa-miR-1178; hsa-miR-626; hsa-miR-148a; hsa-miR-1182
	AREG	374	hsa-miR-517a ; hsa-miR-34c-5p ; hsa-miR-4724-3p ; hsa-miR-556-5p ; hsa-miR-517b
	EREG	2069	hsa-miR-4713-5p ; hsa-miR-4645-5p ; hsa-miR-130a ; hsa-miR-3661 ; hsa-miR-192
	HBEGF	1839	hsa-miR-4736; hsa-miR-1207-5p; hsa-miR-4710; hsa-miR-3160-5p; hsa-miR-1271
	BTC	685	hsa-miR-4715-3p; hsa-miR-1200; hsa-miR-4661-5p; hsa-miR-934; hsa-miR-488
	NRG1	3084	hsa-miR-4632; hsa-miR-1203; hsa-miR-552; hsa-miR-4736; hsa-miR-183
	NRG2	9542	hsa-miR-3196; hsa-miR-3934; hsa-miR-4746-5p; hsa-miR-296-5p; hsa-miR-4665-5p
	NRG4	145957	hsa-miR-608; hsa-miR-1301; hsa-miR-4704-3p; hsa-miR-516b; hsa-miR-3681;
	EGFR	1956	hsa-miR-4417; hsa-miR-608; hsa-miR-885-3p; hsa-miR-4474-3p; hsa-miR-7;
	ERBB2	2064	hsa-miR-331-3p; hsa-miR-4650-5p; hsa-miR-1972; hsa-miR-4533; hsa-miR-1296;
	ERBB3	2065	hsa-miR-3199; hsa-miR-4505; hsa-miR-1287; hsa-miR-3153; hsa-miR-4290;
	ERBB4	2066	hsa-miR-4469; hsa-miR-193a-3p; hsa-miR-642a; hsa-miR-3907; hsa-miR-3187-3p;
CDK4,6	CDK4	1019	hsa-miR-4747-5p; hsa-miR-198; hsa-miR-4728-5p; hsa-miR-765; hsa-miR-4280;
	CDK6	1021	hsa-miR-3680; hsa-miR-3158-3p; hsa-miR-621; hsa-miR-644; hsa-miR-4252;
	CCND1	595	hsa-miR-4707-3p; hsa-miR-3170; hsa-miR-1193; hsa-miR-4740-3p; hsa-miR-4632;
	CCND2	894	hsa-miR-1468; hsa-miR-103b; hsa-miR-1205; hsa-miR-3065-3p; hsa-miR-4718;
	CCND3	896	hsa-miR-4701-5p; hsa-miR-4739; hsa-miR-138; hsa-miR-4749-5p; hsa-miR-3154;
	CDKN2A,	1029	hsa-miR-663b; hsa-miR-675; hsa-miR-663; hsa-miR-1291; hsa-miR-621;
	CDKN2B	1030	hsa-miR-4308; hsa-miR-718; hsa-miR-1914; hsa-miR-451; hsa-miR-346;
	CCNE1	898	hsa-miR-16; hsa-miR-874; hsa-miR-146b-3p; hsa-miR-4524; hsa-miR-3190;
	CCNE2	9134	hsa-miR-449a; hsa-miR-370; hsa-miR-4460; hsa-miR-30b; hsa-miR-485-5p;
	RB1	5925	hsa-miR-4703-5p; hsa-miR-4801; hsa-miR-4432; hsa-miR-7; hsa-miR-525-5p;
PLK / AURK / Kinesins	PLK1	5347	hsa-miR-296-5p; hsa-miR-4660; hsa-miR-3665; hsa-miR-3166; hsa-miR-4778-5p;
	AURKA	6790	hsa-miR-3941; hsa-miR-4655-5p; hsa-miR-4756-5p; hsa-miR-3616-3p; hsa-miR-4757-5p;
	BORA	79866	hsa-miR-532-3p; hsa-miR-3162-3p; hsa-miR-4713-5p; hsa-miR-4758-3p; hsa-miR-3189-5p;
	ILK	3611	hsa-miR-1908; hsa-miR-4505; hsa-miR-744; hsa-miR-4425; hsa-miR-3150a-3p;
	KIF11	3832	
ANGIOGENESIS	VEGFA	7422	hsa-miR-3668; hsa-miR-939; hsa-miR-29a; hsa-miR-339-5p; hsa-miR-16;
	VEGFB	7423	hsa-miR-2467-3p; hsa-miR-4649-3p; hsa-miR-4687-3p; hsa-miR-193a-5p; hsa-miR-1275;
	VEGFC	7424	hsa-miR-711; hsa-miR-3688-5p; hsa-miR-4687-3p; hsa-miR-128; hsa-miR-4318;
	VEGFD	2277	hsa-miR-320e; hsa-miR-135a; hsa-miR-7; hsa-miR-1184; hsa-miR-513b;
	FLT1	2321	hsa-miR-148a; hsa-miR-5095; hsa-miR-335; hsa-miR-615-3p; hsa-miR-149;
	KDR	3791	hsa-miR-4435; hsa-miR-665; hsa-miR-370; hsa-miR-136; hsa-miR-138;
	FLT4	2324	hsa-miR-4707-3p; hsa-miR-2861; hsa-miR-4728-5p; hsa-miR-2467-3p; hsa-miR-4783-5p;
	PDGFA	5154	hsa-miR-4690-5p; hsa-miR-3917; hsa-miR-4706; hsa-miR-4768-5p; hsa-miR-412;

	PDGFB	5155	hsa-miR-3202; hsa-miR-1909; hsa-miR-3689d; hsa-miR-4271; hsa-miR-625;
	PDGFRA	5156	hsa-miR-3691-3p; hsa-miR-4471; hsa-miR-34a; hsa-miR-663b; hsa-miR-3117-3p;
	PDGFRB	5159	hsa-miR-1915; hsa-miR-4292; hsa-miR-4731-5p; hsa-miR-637; hsa-miR-486-3p;
	Kit	3815	hsa-miR-4254; hsa-miR-671-5p; hsa-miR-1193; hsa-miR-222; hsa-miR-4485;
**ANGIOPOIETINS**	THBS1	7057	hsa-miR-3074-5p; hsa-miR-4786-3p; hsa-miR-3177-5p; hsa-miR-634; hsa-miR-4443;
	TGFB1	7040	hsa-miR-3196; hsa-miR-663; hsa-miR-296-5p; hsa-miR-3943; hsa-miR-3183;
	ANGPT1	284	hsa-miR-153; hsa-miR-4643; hsa-miR-4755-5p; hsa-miR-4789-3p; hsa-miR-3682-3p;
	ANGPT2	285	hsa-miR-135a; hsa-miR-1182; hsa-miR-513c; hsa-miR-597; hsa-miR-4251;
	ANGPTL1	9068	hsa-miR-3688-5p; hsa-miR-586; hsa-miR-4480; hsa-miR-544; hsa-miR-194;
	ANGPT4	51378	hsa-miR-296-5p; hsa-miR-4690-3p; hsa-miR-422a; hsa-miR-431; hsa-miR-665;
	TIE1	7075	hsa-miR-3151; hsa-miR-4447; hsa-miR-4723-5p; hsa-miR-486-3p; hsa-miR-4287;
	TEK	7010	hsa-miR-4713-5p; hsa-miR-300; hsa-miR-4690-3p; hsa-miR-150; hsa-miR-148a;
**IMMUNO-Modulator**	CD274 or PD1L	29126	hsa-miR-4443; hsa-miR-3117-3p; hsa-miR-138; hsa-miR-339-5p; hsa-miR-1273;
	PDCD1LG2	80380	hsa-miR-20a; hsa-miR-548an; hsa-miR-4661-5p; hsa-miR-3133; hsa-miR-3910;
	PDCD1	5133	hsa-miR-4290; hsa-miR-1291; hsa-miR-4763-5p; hsa-miR-2861; hsa-miR-661;
	CTLA4	1493	hsa-miR-324-5p; hsa-miR-502-5p; hsa-miR-4254; hsa-miR-3121-5p; hsa-miR-1587;
	LAG3	3902	hsa-miR-4515; hsa-miR-1269; hsa-miR-4529-3p; hsa-miR-4270; hsa-miR-628-5p;
**PI3K**	PIK3CA	5290	hsa-miR-4450; hsa-miR-4529-3p; hsa-miR-302d; hsa-miR-3910; hsa-miR-490-5p;
	PIK3CB	5291	
	PIK3CD	5293	hsa-miR-4537; hsa-miR-2355-5p; hsa-miR-523; hsa-miR-7; hsa-miR-484;
	PIK3CG	5294	hsa-miR-370; hsa-miR-3135b; hsa-miR-1976; hsa-miR-1276; hsa-miR-3672;
	PIK3C2B	5287	hsa-miR-361-3p; hsa-miR-4728-5p; hsa-miR-4740-3p; hsa-miR-3612; hsa-miR-4314;
	PRKCB	5579	hsa-miR-4691-5p; hsa-miR-448; hsa-miR-7; hsa-miR-668; hsa-miR-27a;
	PRKCA	5578	hsa-miR-4757-5p; hsa-miR-4685-5p; hsa-miR-4706; hsa-miR-1275; hsa-miR-4525;
	PIK3R1	5295	hsa-miR-4789-3p; hsa-miR-4789-5p; hsa-miR-4646-3p; hsa-miR-1184; hsa-miR-4660;
	PIK3R2	5296	hsa-miR-4723-5p; hsa-miR-3180; hsa-miR-4447; hsa-miR-3960; hsa-miR-3151;
	PIK3R3	8503	hsa-miR-4725-3p; hsa-miR-4435; hsa-miR-4715-5p; hsa-miR-2115; hsa-miR-4313;
**MET**	HGF	3082	hsa-miR-4520a-3p; hsa-miR-764; hsa-miR-4716-3p; hsa-miR-1288; hsa-miR-4710;
	MET	4233	hsa-miR-3074-5p; hsa-miR-2682; hsa-miR-34c-5p; hsa-miR-182; hsa-miR-1269b;
	AXL	558	hsa-miR-3142; hsa-miR-4728-5p; hsa-miR-924; hsa-miR-3689c; hsa-miR-432;
	MST1R	4486	hsa-miR-296-5p; hsa-miR-218; hsa-miR-1286; hsa-miR-3126-5p; hsa-miR-4284;
**MEK**	MAP2K1	5604	hsa-miR-4323; hsa-miR-4423-3p; hsa-miR-758; hsa-miR-34a; hsa-miR-15b;
	MAP2K2	5605	hsa-miR-1181; hsa-miR-1207-3p; hsa-miR-744; hsa-miR-663; hsa-miR-4786-5p;
	MAP2K3	5606	hsa-miR-4313; hsa-miR-3151; hsa-miR-4283; hsa-miR-4540; hsa-miR-4270;
	MAP2K4	6416	hsa-miR-4663; hsa-miR-25; hsa-miR-3065-3p; hsa-miR-4649-5p; hsa-miR-627;
	MAP3K1	4214	hsa-miR-4286; hsa-miR-1225-3p; hsa-miR-4703-3p; hsa-miR-544; hsa-miR-887;
	MAP3K2	10746	hsa-miR-519d; hsa-miR-651; hsa-miR-587; hsa-miR-34c-3p; hsa-miR-2909;
	MAP3K3	4215	hsa-miR-661; hsa-miR-1225-3p; hsa-miR-544b; hsa-miR-3922-3p; hsa-miR-4505;
	MAP3K4	4216	hsa-miR-1204; hsa-miR-3129-5p; hsa-miR-5047; hsa-miR-3691-3p; hsa-miR-3064-3p;
**ERK**	MAPK3	5595	hsa-miR-4270; hsa-miR-486-3p; hsa-miR-483-5p; hsa-miR-608; hsa-miR-1291;
	MAPK1	5594	hsa-miR-4667-5p; hsa-miR-4459; hsa-miR-4271; hsa-miR-4799-5p; hsa-miR-2110;
	KSR1	8844	hsa-miR-331-3p; hsa-miR-4440; hsa-miR-4291; hsa-miR-4660; hsa-miR-876-3p;
	MAPK11	5600	hsa-miR-4640-3p; hsa-miR-296-5p; hsa-miR-4292; hsa-miR-4532; hsa-miR-4685-5p;
**ANTI-APOPTOSIS**	BCL2	596	hsa-miR-448; hsa-miR-4691-3p; hsa-miR-3199; hsa-miR-3943; hsa-miR-342-3p;

	BCL2L1	598	hsa-miR-4447; hsa-miR-608; hsa-miR-4728-5p; hsa-miR-4649-3p; hsa-miR-4700-5p;
	BIRC5	332	hsa-miR-542-3p; hsa-miR-3940-3p; hsa-miR-4660; hsa-miR-1225-3p; hsa-miR-1273;
	XIAP	331	hsa-miR-377; hsa-miR-3150a-3p; hsa-miR-3175; hsa-miR-5095; hsa-miR-3664-5p;
	BAK1	578	hsa-miR-4419a; hsa-miR-125b; hsa-miR-4667-5p; hsa-miR-1909; hsa-miR-4739;
**FGF**	FGF1	2246	hsa-miR-4297; hsa-miR-3155; hsa-miR-1909; hsa-miR-566; hsa-miR-2355-5p;
	FGF2	2247	hsa-miR-195; hsa-miR-4524; hsa-miR-503; hsa-miR-646; hsa-miR-3607-5p;
	FGF3	2248	hsa-miR-3173-5p; hsa-miR-4487; hsa-miR-760; hsa-miR-4722-3p; hsa-miR-4758-3p;
	FGF4	2249	hsa-miR-4671-5p; hsa-miR-3679-3p; hsa-miR-4290; hsa-miR-361-3p; hsa-miR-767-5p;
	FGF5	2250	hsa-miR-4435; hsa-miR-4655-5p; hsa-miR-4288; hsa-miR-4463; hsa-miR-4704-3p;
	FGF6	2251	hsa-miR-4677-3p; hsa-miR-548q; hsa-miR-138; hsa-miR-639; hsa-miR-1322;
	FGF7	2252	hsa-miR-4762-5p; hsa-miR-486-5p; hsa-miR-195; hsa-miR-3920; hsa-miR-1253;
	FGF8	2253	hsa-miR-3120-3p; hsa-miR-545; hsa-miR-491-5p; hsa-miR-361-3p; hsa-miR-4720-5p;
	FGF9	2254	hsa-miR-1273c; hsa-miR-140-5p; hsa-miR-423-3p; hsa-miR-3157-5p; hsa-miR-3683;
	FGF10	2255	
	FGF11	2256	hsa-miR-4667-3p; hsa-miR-4469; hsa-miR-3192; hsa-miR-3661; hsa-miR-3649;
	FGF12	2257	hsa-miR-4747-5p; hsa-miR-3202; hsa-miR-4533; hsa-miR-4633-3p; hsa-miR-197;
	FGF13	2258	hsa-miR-1262; hsa-miR-3675-5p; hsa-miR-1185; hsa-miR-512-3p; hsa-miR-4421;
	FGF14	2259	hsa-miR-4663; hsa-miR-188-3p; hsa-miR-4299; hsa-miR-4690-5p; hsa-miR-4691-3p;
	FGFR1	2260	hsa-miR-4530; hsa-miR-4728-5p; hsa-miR-515-3p; hsa-miR-1208; hsa-miR-4667-5p;
	FGFR2	2263	hsa-miR-515-5p; hsa-miR-3177-3p; hsa-miR-423-3p; hsa-miR-4789-3p; hsa-miR-3675-5p;
	FGFR3	2261	hsa-miR-296-5p; hsa-miR-4793-3p; hsa-miR-4746-3p; hsa-miR-3918; hsa-miR-1291;
	FGFR4	2264	hsa-miR-3177-3p; hsa-miR-4726-5p; hsa-miR-1225-3p; hsa-miR-378g; hsa-miR-564;
**mTOR -AKT-PTEN-**	mTor	2475	hsa-miR-767-3p; hsa-miR-4762-3p; hsa-miR-496; hsa-miR-1233; hsa-miR-1229;
	AKT1	207	hsa-miR-1915; hsa-miR-4721; hsa-miR-3162-3p; hsa-miR-4738-5p; hsa-miR-4723-5p;
	AKT2	208	hsa-miR-4716-3p; hsa-miR-29b; hsa-miR-4278; hsa-miR-3943; hsa-miR-3065-3p;
	PTEN	5728	hsa-miR-642b; hsa-miR-486-5p; hsa-miR-148a; hsa-miR-3944-5p; hsa-miR-3691-5p;
**Modulators MTKPT**	TSC1	7248	hsa-miR-130a; hsa-miR-1537; hsa-miR-637; hsa-miR-3141; hsa-miR-3684;
	TSC2	7249	hsa-miR-4420; hsa-miR-654-3p; hsa-miR-4722-5p; hsa-miR-615-5p; hsa-miR-3922-5p;
	STK11	6794	hsa-miR-663; hsa-miR-744; hsa-miR-4723-5p; hsa-miR-3960; hsa-miR-615-5p;
	PIM1	5292	hsa-miR-4749-3p; hsa-miR-761; hsa-miR-3689a-3p; hsa-miR-331-3p; hsa-miR-4436b-3p;
	PIM2	11040	hsa-miR-361-3p; hsa-miR-4532; hsa-miR-3654; hsa-miR-4645-5p; hsa-miR-4768-3p;
	PIM3	415116	hsa-miR-3195; hsa-miR-4697-5p; hsa-miR-654-5p; hsa-miR-4467; hsa-miR-637;
**RAS**	KRAS	3845	hsa-miR-3923; hsa-miR-4323; hsa-miR-4447; hsa-miR-513a-5p; hsa-miR-548ag;
	NRAS	4893	hsa-miR-502-5p; hsa-miR-1296; hsa-miR-1324; hsa-miR-3120-3p; hsa-miR-4271;
	HRAS	3265	hsa-miR-3667-3p; hsa-miR-4728-5p; hsa-miR-4292; hsa-miR-4532; hsa-miR-663;
**RAF**	RAF1	5894	hsa-miR-1291; hsa-miR-7; hsa-miR-3126-5p; hsa-miR-296-5p; hsa-miR-764;
	BRAF	673	hsa-miR-617; hsa-miR-2110; hsa-miR-3977; hsa-miR-1182; hsa-miR-1289;
**TELOMERASE**	TERT	7015	hsa-miR-4650-5p; hsa-miR-491-5p; hsa-miR-4651; hsa-miR-3687; hsa-miR-4292;
	TERC	7012	
	TEP1	7011	hsa-miR-1911; hsa-miR-3132; hsa-miR-136; hsa-miR-2861; hsa-miR-31;
	HSP90AA1	3320	hsa-miR-4753-5p; hsa-miR-632; hsa-miR-519e; hsa-miR-3679-3p; hsa-miR-134;
	DKC1	1736	hsa-miR-3194-3p; hsa-miR-621; hsa-miR-3620; hsa-miR-646; hsa-miR-4279;
	PTGES3	10728	hsa-miR-3189-5p; hsa-miR-3135; hsa-miR-4266; hsa-miR-3678-3p; hsa-miR-4286;

IGF & Warburg	IGF1	3479	hsa-miR-483-3p; hsa-miR-1275; hsa-miR-4435; hsa-miR-488; hsa-miR-625;
	IGF2	3481	hsa-miR-4447; hsa-miR-491-5p; hsa-miR-210; hsa-miR-3191; hsa-miR-3144-5p;
	IGF1R	3480	hsa-miR-4746-3p; hsa-miR-4784; hsa-miR-4763-3p; hsa-miR-4327; hsa-miR-3157-5p;
	IGF2R	3482	hsa-miR-4667-3p; hsa-miR-653; hsa-miR-4707-3p; hsa-miR-4736; hsa-miR-548an;
	INSR	3643	hsa-miR-2467-5p; hsa-miR-3975; hsa-miR-3188; hsa-miR-4707-3p; hsa-miR-4290;
	IRS1	3667	hsa-miR-660; hsa-miR-541; hsa-miR-4462; hsa-miR-544b; hsa-miR-183;
	PKM2	5315	hsa-miR-762; hsa-miR-625; hsa-miR-612; hsa-miR-4675; hsa-miR-4665-5p;
WNT	CDH1	999	hsa-miR-4640-3p; hsa-miR-4711-5p; hsa-miR-3689c; hsa-miR-2355-5p; hsa-miR-1296;
	CTNNA1	1495	hsa-miR-1288; hsa-miR-4440; hsa-miR-4515; hsa-miR-4705; hsa-miR-9;
	CTNNB1	1499	hsa-miR-3688-5p; hsa-miR-3162-3p; hsa-miR-4776-5p; hsa-miR-4496; hsa-miR-3619-3p;
	WNT 1	7471	hsa-miR-4488; hsa-miR-4784; hsa-miR-4695-5p; hsa-miR-4644; hsa-miR-4689;
	FZD1	8321	hsa-miR-4269; hsa-miR-4769-5p; hsa-miR-1275; hsa-miR-1324; hsa-miR-4279;
	WNT5A	7474	hsa-miR-2110; hsa-miR-4691-5p; hsa-miR-876-5p; hsa-miR-3127-3p; hsa-miR-4656;
	WNT5B	81029	hsa-miR-4316; hsa-miR-4258; hsa-miR-2909; hsa-miR-1296; hsa-miR-486-3p;
	FZD5	7855	hsa-miR-296-5p; hsa-miR-3943; hsa-miR-188-3p; hsa-miR-3661; hsa-miR-3672;
	WIF1	11197	hsa-miR-1972; hsa-miR-3938; hsa-miR-548v; hsa-miR-140-3p; hsa-miR-3977;
	DKK1	22943	hsa-miR-493; hsa-miR-4639-3p; hsa-miR-4727-5p; hsa-miR-4678; hsa-miR-934;
PARP	PARP1	142	hsa-miR-891b; hsa-miR-4536; hsa-miR-4451; hsa-miR-555; hsa-miR-7;
	BRCA1	672	hsa-miR-615-5p; hsa-miR-3667-3p; hsa-miR-4446-3p; hsa-miR-760; hsa-miR-4656;
	XRCC1	7515	hsa-miR-589; hsa-miR-4477a;
	RAD54L	8438	hsa-miR-4713-5p; hsa-miR-1225-3p; hsa-miR-3918; hsa-miR-3667-3p; hsa-miR-1291;
	RAD54B	25788	hsa-miR-587; hsa-miR-4268; hsa-miR-548s; hsa-miR-3926; hsa-miR-1;
	ATM	472	hsa-miR-892b; hsa-miR-193a-3p; hsa-miR-4735-3p; hsa-miR-4736; hsa-miR-4262;
	ATR	545	hsa-miR-3613-5p; hsa-miR-383; hsa-miR-4760-5p; hsa-miR-140-3p; hsa-miR-586;
	CHEK1	1111	hsa-miR-2355-5p; hsa-miR-541; hsa-miR-1286; hsa-miR-4733-3p; hsa-miR-16;
	CHEK2	11200	hsa-miR-3118; hsa-miR-759; hsa-miR-4276; hsa-miR-3938; hsa-miR-943;
	WEE1	7465	hsa-miR-4716-3p; hsa-miR-4723-5p; hsa-miR-424; hsa-miR-3120-3p; hsa-miR-4278;
HDAC	HDAC1	3065	hsa-miR-4284; hsa-miR-4292; hsa-miR-4271; hsa-miR-3126-5p; hsa-miR-584;
	HDAC2	3066	hsa-miR-362-5p; hsa-miR-3977; hsa-miR-3194-3p; hsa-miR-4662a-5p; hsa-miR-4720-5p;
	HDAC3	8841	hsa-miR-3189-3p; hsa-miR-1261; hsa-miR-326; hsa-miR-1302; hsa-miR-4308;
	HDAC4	9759	hsa-miR-4292; hsa-miR-4313; hsa-miR-4728-5p; hsa-miR-1225-3p; hsa-miR-4316;
	HDAC5	10014	hsa-miR-331-3p; hsa-miR-671-5p; hsa-miR-4498; hsa-miR-296-5p; hsa-miR-4505;
JAK-STAT	JAK1	3716	hsa-miR-4252; hsa-miR-4437; hsa-miR-4520a-3p; hsa-miR-323b-5p; hsa-miR-4674;
	JAK2	3717	hsa-miR-4720-5p; hsa-miR-4468; hsa-miR-3120-3p; hsa-miR-4777-3p; hsa-miR-568;
	STAT1	6772	hsa-miR-4682; hsa-miR-1252; hsa-miR-3119; hsa-miR-4697-3p; hsa-miR-2682;
	STAT2	6773	hsa-miR-665; hsa-miR-3202; hsa-miR-4292; hsa-miR-4313; hsa-miR-1289;
	STAT3	6774	hsa-miR-1299; hsa-miR-4753-5p; hsa-miR-1184; hsa-miR-874; hsa-miR-5047;
	SOCS1	8651	hsa-miR-4645-5p; hsa-miR-556-3p; hsa-miR-331-3p; hsa-miR-4716-3p; hsa-miR-324-5p;
HEDGEHOG	SHH	6469	hsa-miR-1471; hsa-miR-4749-3p; hsa-miR-4313;
	PTCH1	5727	hsa-miR-4757-5p; hsa-miR-564; hsa-miR-1262; hsa-miR-767-3p; hsa-miR-125a-3p;
	SMO	6608	hsa-miR-370; hsa-miR-4690-3p; hsa-miR-4758-3p; hsa-miR-423-3p; hsa-miR-1915;
	STK36	27148	hsa-miR-571; hsa-miR-3192; hsa-miR-581; hsa-miR-920; hsa-miR-4715-5p;
	PRKACA	5566	hsa-miR-4723-5p; hsa-miR-4665-5p; hsa-miR-608; hsa-miR-423-5p; hsa-miR-625;

	SUFU	51684	hsa-miR-3184; hsa-miR-4487; hsa-miR-4688; hsa-miR-4728-5p; hsa-miR-4741;
	GLI1	2735	hsa-miR-3943; hsa-miR-4279; hsa-miR-4292; hsa-miR-361-3p; hsa-miR-4533;
**DNA REPAIR**	ERCC1	2067	hsa-miR-661; hsa-miR-1913; hsa-miR-323-5p; hsa-miR-1972; hsa-miR-1268;
	RAD52	5893	hsa-miR-3922-3p; hsa-miR-4725-3p; hsa-miR-342-3p; hsa-miR-542-3p; hsa-miR-4303;
	XRCC4	7518	hsa-miR-361-5p; hsa-miR-380; hsa-miR-4520a-3p; hsa-miR-3121-5p; hsa-miR-2355-3p;
	RAD51	5888	hsa-miR-198; hsa-miR-532-3p; hsa-miR-606; hsa-miR-4430; hsa-miR-4432;
	BRCA1	672	hsa-miR-615-5p; hsa-miR-3667-3p; hsa-miR-4446-3p; hsa-miR-760; hsa-miR-4656;
	NEDD8	4738	hsa-miR-4713-3p; hsa-miR-4726-5p; hsa-miR-665; hsa-miR-1285; hsa-miR-1322;
	NAE1	8883	hsa-miR-4524; hsa-miR-646; hsa-miR-4660; hsa-miR-582-5p; hsa-miR-603;
**NOTCH**	NOTCH1	4851	hsa-miR-4313; hsa-miR-4268; hsa-miR-449a; hsa-miR-139-5p; hsa-miR-4727-5p;
	Adam17	6868	hsa-miR-507; hsa-miR-3918; hsa-miR-4687-5p; hsa-miR-3651; hsa-miR-1827;
	PSEN1	5663	hsa-miR-3065-3p; hsa-miR-4697-3p; hsa-miR-3120-5p; hsa-miR-4303; hsa-miR-488;
	NCSTN	23385	hsa-miR-339-5p; hsa-miR-4654; hsa-miR-1321; hsa-miR-4648; hsa-miR-3657;
	JAG1	182	hsa-miR-4692; hsa-miR-1273g; hsa-miR-920; hsa-miR-4661-5p; hsa-miR-4283;
	SRRT	51593	hsa-miR-4700-3p; hsa-miR-3190; hsa-miR-487b; hsa-miR-520f; hsa-miR-3929;
	APH1A	51107	hsa-miR-3679-3p; hsa-miR-198; hsa-miR-3173-3p; hsa-miR-4685-5p; hsa-miR-3131;
**Others**	ROS1	6098	hsa-miR-4693-3p; hsa-miR-4653-3p; hsa-miR-33a; hsa-miR-606; hsa-miR-3659;
	ALK	238	hsa-miR-642a; hsa-miR-646; hsa-miR-4764-3p; hsa-miR-1271; hsa-miR-4713-3p;
	RET	5979	hsa-miR-544; hsa-miR-4652-5p; hsa-miR-510; hsa-miR-31; hsa-miR-3622b-5p;
	UBA1	7317	hsa-miR-4716-3p; hsa-miR-762; hsa-miR-4640-5p; hsa-miR-3202; hsa-miR-31;

Supplemental Table 5. *Summary of the scores obtained for all patients of the 121 NSCLC, for a selection of interventional points described in Table 1*

patient	Histo	Her	CDK 4_6	ANGIO	PI3K	MET	MEK	ERK	FGF	mTOR	RAS	RAF	PARP	JAK_STAT	PD1L	CTLA4
1	AC	1	3	5	2	4	9	3	5	5	6	3	8	6	9	9
2	AC	5	6	7	7	10	2	1	3	2	7	4	5	8	10	9
3	SCC	9	4	1	1	1	3	1	8	2	3	2	8	2	1	4
4	AC	2	2	5	7	2	8	9	9	7	10	10	5	8	9	2
5	AC	8	10	9	4	7	7	5	2	3	2	9	3	9	2	10
6	SCC	8	7	10	10	6	4	7	9	8	8	8	7	7	7	10
7	AC	10	1	3	1	3	5	2	1	4	4	5	6	5	5	3
8	AC	5	10	8	3	9	6	5	2	1	6	5	4	5	10	8
9	SCC	5	1	1	6	5	4	6	6	2	5	4	1	8	5	7
10	SCC	1	1	2	4	5	8	4	2	3	9	6	2	7	10	5
11	LCC	7	3	9	5	5	7	3	6	2	10	10	5	6	8	8
12	AC	7	2	4	2	3	3	4	6	7	7	3	2	1	4	2
13	SCC	10	9	7	7	5	7	6	9	7	10	4	9	8	3	8
14	AC	9	3	6	2	5	8	2	7	5	6	4	3	6	3	6
15	SCC	2	9	1	10	9	3	2	5	7	1	1	10	3	4	1
16	AC	6	5	5	5	6	2	9	8	7	1	6	5	4	6	10
17	AC	4	8	8	2	7	9	7	1	2	2	5	6	10	6	10
18	AC	10	9	9	8	10	10	5	6	10	2	1	8	7	10	8
19	Other	10	9	6	8	10	9	6	7	4	2	1	1	10	3	7
20	LCC	1	4	1	3	10	2	4	2	5	3	10	9	2	2	4
21	AC	8	5	2	8	9	4	10	1	3	4	5	3	5	1	4
22	LCC	8	5	5	1	6	2	6	2	1	3	2	5	3	5	6
23	LCC	4	6	10	3	9	7	10	6	10	3	7	2	5	7	9
24	AC	6	9	1	2	8	6	8	3	9	3	9	10	9	6	7
25	AC	2	10	1	10	4	5	10	7	8	6	10	10	1	1	5
26	SCC	6	10	10	6	8	6	1	10	10	9	5	4	7	9	9
27	SCC	2	10	9	7	2	10	3	3	6	10	9	4	9	9	10
28	SCC	1	5	10	2	7	3	5	10	4	2	7	6	2	7	7
29	AC	7	8	7	10	10	9	4	3	9	10	7	4	10	9	10
30	SCC	5	9	6	8	6	6	10	8	10	2	6	9	2	4	3
31	AC	10	6	3	7	9	7	8	6	8	5	8	10	10	8	7
32	AC	5	4	6	4	7	3	4	3	3	6	6	2	2	5	5
33	Other	9	6	3	6	10	4	1	9	5	9	10	6	6	5	5
34	SCC	3	10	3	9	7	4	10	8	5	8	4	10	4	10	5
35	SCC	6	5	10	4	4	8	9	7	9	2	8	10	6	10	4
36	AC	3	10	8	1	8	2	7	9	10	8	8	3	3	5	1
37	SCC	7	5	2	9	8	7	4	3	9	7	5	9	5	4	7
38	LCC	4	8	4	1	1	3	2	10	5	9	10	4	3	5	2
39	AC	10	3	6	7	7	5	3	2	1	7	6	10	7	6	9
40	AC	4	2	8	2	4	3	4	3	8	4	6	7	2	7	5
41	SCC	3	5	7	6	5	7	2	6	6	5	5	1	4	3	3
42	AC	10	10	9	6	7	10	3	1	4	3	10	3	10	10	8
43	AC	5	5	8	1	2	2	5	5	8	1	1	10	1	3	2
44	AC	7	6	9	6	10	5	7	4	4	1	8	1	7	4	6
45	SCC	6	7	4	10	3	10	6	3	7	6	4	6	8	10	6
46	AC	2	2	3	5	6	9	8	1	3	6	8	2	3	5	7

47	SCC	10	8	4	10	9	6	5	6	6	1	7	8	1	1	3
48	SCC	6	7	6	9	6	1	10	5	4	4	1	10	6	8	6
49	SCC	10	7	3	8	4	4	4	2	4	2	4	10	7	8	8
50	AC	10	7	8	10	3	9	4	8	10	9	4	4	5	9	10
51	SCC	3	6	2	5	10	5	10	4	1	10	6	7	8	4	7
52	AC	7	7	10	3	8	10	1	3	6	10	9	5	5	3	4
53	SCC	3	1	2	6	6	6	3	9	7	7	3	6	6	6	6
54	AC	9	1	3	4	7	4	10	4	4	8	9	2	2	1	3
55	AC	10	4	7	4	8	1	5	4	2	7	3	3	4	4	2
56	SCC	9	4	6	9	7	1	2	10	4	6	3	8	1	1	1
57	AC	10	9	7	10	6	8	8	4	8	10	10	8	10	8	9
58	AC	9	6	4	10	9	2	3	2	1	6	7	1	9	2	7
59	AC	10	7	3	7	3	6	3	10	6	10	10	1	8	2	4
60	AC	9	2	3	9	9	10	5	10	8	8	10	2	6	2	6
61	SCC	3	2	10	5	1	10	10	9	1	9	1	5	8	3	1
62	SCC	10	6	6	10	3	8	1	6	10	1	2	9	2	3	2
63	AC	8	3	10	6	8	9	10	8	10	5	9	5	10	4	6
64	AC	1	7	9	5	4	3	6	10	9	3	8	7	3	4	1
65	SCC	3	4	2	4	2	1	6	5	5	8	4	6	9	3	4
66	SCC	1	2	5	7	1	7	7	1	2	8	7	1	9	6	5
67	AC	7	1	8	4	2	1	2	1	5	9	3	1	4	8	6
68	LCC	10	1	3	10	9	8	6	8	8	6	10	5	9	10	5
69	SCC	4	4	9	8	9	8	4	7	10	6	4	2	4	9	9
70	AC	9	8	10	1	1	2	2	1	9	1	4	3	1	2	1
71	AC	10	1	8	7	9	7	10	8	8	4	8	1	10	4	9
72	AC	6	7	10	5	4	5	5	4	4	9	7	5	3	3	4
73	LCC	10	8	1	4	10	9	1	8	9	5	10	10	4	8	10
74	SCC	10	9	1	9	3	8	3	9	3	9	7	7	10	10	10
75	SCC	8	5	10	3	8	1	9	8	8	1	2	5	3	5	1
76	SCC	7	9	8	1	2	3	9	10	10	1	2	9	2	2	3
77	SCC	1	2	5	3	5	2	7	1	1	3	2	4	8	7	4
78	AC	1	10	1	2	10	2	8	5	9	1	1	6	7	2	9
79	LCC	9	8	4	6	3	2	6	1	10	1	2	10	1	1	1
80	SCC	3	3	1	3	6	1	10	10	9	6	1	7	5	1	3
81	AC	8	2	10	8	7	3	1	3	2	5	6	3	1	2	6
82	SCC	2	1	10	5	3	3	2	5	3	9	7	4	6	6	8
83	AC	7	1	8	1	1	5	8	2	2	4	5	7	7	6	4
84	SCC	4	4	4	2	7	10	8	5	7	5	9	3	2	7	1
85	SCC	5	2	5	2	1	6	7	10	5	5	7	4	2	3	4
86	SCC	7	9	4	3	4	2	10	9	8	7	9	7	7	4	8
87	AC	9	4	8	6	3	5	6	5	3	2	7	7	5	6	7
88	SCC	7	6	5	10	6	6	9	7	8	1	9	8	9	6	5
89	LCC	3	5	7	2	5	8	7	5	7	5	5	10	9	9	9
90	SCC	4	10	2	10	4	5	7	7	10	5	4	1	7	4	8
91	AC	10	2	6	8	8	8	2	3	3	10	6	3	4	9	4
92	AC	10	8	2	8	8	10	2	2	9	7	10	6	10	5	6
93	AC	10	8	7	3	10	9	5	4	3	4	10	2	8	7	7
94	AC	6	1	9	3	2	1	9	4	1	3	1	1	1	1	1
95	LCC	1	9	5	7	1	10	3	1	9	2	3	7	3	1	2
96	AC	10	2	5	6	4	1	5	4	2	3	9	2	4	2	2
97	Other	1	4	2	1	2	1	7	9	1	4	6	4	8	8	3
98	AC	2	3	7	4	6	8	1	4	5	7	8	2	6	7	8
99	SCC	5	1	9	1	1	7	7	2	6	4	2	4	1	5	2
100	SCC	6	8	5	10	2	4	6	9	2	8	5	8	2	2	2

101	AC	9	8	7	3	5	9	1	7	6	4	5	4	3	8	3
102	LCC	4	9	6	8	3	10	9	6	10	3	3	9	1	8	5
103	SCC	10	6	4	9	5	7	9	9	6	2	2	8	10	6	10
104	AC	10	10	4	9	9	4	9	6	1	10	8	6	10	10	10
105	SCC	2	6	7	7	3	9	7	5	4	7	7	9	6	5	9
106	AC	5	8	10	9	8	4	5	10	7	8	1	1	6	8	3
107	SCC	3	5	2	2	1	5	9	10	1	8	5	7	1	1	1
108	LCC	2	4	10	1	5	7	9	4	2	5	9	2	5	7	2
109	AC	8	3	8	5	10	5	8	2	4	10	6	3	5	6	7
110	SCC	4	3	9	7	4	6	2	5	7	4	3	9	5	7	5
111	AC	10	5	5	3	2	4	8	1	3	8	1	9	3	7	1
112	SCC	3	7	3	4	1	1	8	8	1	2	3	8	3	2	3
113	AC	9	6	10	5	10	3	1	7	6	10	2	5	4	3	6
114	SCC	2	3	7	4	5	4	1	7	5	8	3	6	4	8	3
115	SCC	10	4	4	6	7	6	8	7	5	4	6	7	9	10	8
116	SCC	1	10	1	10	1	5	4	4	6	3	1	9	4	1	9
117	SCC	5	3	6	5	2	6	6	8	6	5	9	8	8	9	5
118	SCC	6	10	9	8	8	1	4	6	9	9	2	8	7	9	10
119	SCC	2	3	1	8	2	9	3	7	3	9	3	9	10	10	8
120	AC	2	7	4	9	4	10	3	3	6	7	8	6	9	7	10
121	AC	8	7	10	9	6	10	8	10	7	7	8	3	9	9	2

Supplemental Table 6. Demonstration of the complexity of co-activation of interventional points. *Abbreviations used to designate interventional points described in Supplemental Table 2. Each patient's tumor shows multiple activations, suggesting multiple possibilities of combinations. All 24 interventional points were analyzed. Yellow boxes represent high scores 8, 9 and 10. Blue Boxes represent medium activation (scores 6 and 7)*

ID	High activation score: 8, 9 and 10								Medium activation score: 6 and 7					
1	Antiap	MEK	IGF	PD1L	CTLA4	PARP	AURKA	RAS	JAK_STAT	DNAREP				
2	Antiap	IGF	PD1L	CTLA4	CDK 4_6	SCDK 4_6	RCDK 4_6	ANGIO	PI3K	RAS	WNT	DNAREP	NOTCH	
3	Antiap	CDK 4_6	AURKA	PI3K	ERK	mTKPT	TELOME	IGF_War	WNT	PARP	HDAC	HEDGEH	DNAREP	NOTCH
4	Antiap	CDK 4_6	SCDK 4_6	RCDK 4_6	ANGIO	MET	FGF	mTKPT	ModMTKT	RAS	RAF	TELOME	IGF	HEDGEH
5	Antiap	ANGIO	mTKPT	RAF	TELOME	PARP	PD1L							
6	Antiap	Her	CDK 4_6	ANGIO	MEK	RAF	JAK_STAT	NOTCH	PD1L	CTLA4	AURKA	RAS	JAK_STAT	DNA_REP
7	Antiap	Her	AURKA	ANGIO	AGPT	PI3K	MEK	FGF	mTKPT	ModMTKT	RAS	TELOME	PD11L	CTLA4
8	Antiap	Her	CDK 4_6	ANGIO	mTKPT	PLAURKi	TELOME	HDAC						
9	Antiap	ANGIO	AGPT	RAS	IGF_War	Her	CDK 4_6	SCDK 4_6	RCDK 4_6	RAF				
10	Antiap	Her	RAS	HDAC	PD1L	ANGIO	RAF	IGF_War						
11	Antiapo	Her	ANGIO	PI3K	MEK	ERK	FGF	RAF	IGF_War	WNT	HDAC	JAK_STAT	PD1L	CDK 4_6
12	AGPT	MEK	ERK	FGF	RAS	RAF	JAK_STAT	NOTCH	PD1L	PI3K	mTKPT	WNT	HDAC	
13	Her	CDK 4_6	ANGIO	AGPT	RAF	TELOM	JAK_STAT	CTLA4	MET	MEK	WNT			
14	Her	PARP	HDAC	HEDGEH										
15	CDK 4_6	AURKA	ANGIO	MET	Antiap	TELOM	PD1L	CTLA4	MEK	RAS	HDAC			
16	Her	FGF	mTKPT	RAS	IGF	HEDGEH OG								
17	Her	AGPT	MEK	WNT	ANGIO	FGF	RAS	IGF_War	JAK_STAT	NOTCH	CTLA4			
18	AGPT	FGF	CTLA4	Her	AURKA	MET	Antiap	RAF	HDAC	DNA_REP	PD1L			
19	CDK 4_6	ANGIO	MEK	JAK_STA T	CTLA4									
20	Her	CDK 4_6	AURKA	ANGIO	PI3K	MET	MEK	Antiap	mTKPT	PARP	DNA_REP	PD1L	CTLA4	FGF
21	Her	PI3K	MET	ERK	AGPT	WNT								
22	CDK 4_6	AURKA	MET	ERK	mTKPT	RAF	TELOM	IGF_War	PARP	DNA_REP	NOTCH	Her	MEK	Antiap
23	CDK 4_6	PI3K	MET	MEK	Antiap	mTKPT	M_MTKPT	RAS	TELOM	WNT	JAK_STAT	PD1L	CTLA4	Her
24	Her	AGPT	MET	ERK	mTKPT	RAF	TELOM	PARP	HDAC	JAK_STAT	DNA_REP	PD1L	PI3K	MEK
25	ANGIO	MET	RAS	RAF	IGF_War	PD1L	CTLA4							
26	Antiap	IGF_Wa r	PARP	HDAC	DNA_REP	CTLA4	Her	AURKA	ANGIO	PI3K	MET	RAS	RAF	JAK_STAT
27	ANGIO	AGPT	mTKPT	TELOM	IGF_War	WNT	PARP	HDAC	DNA_REP	PLAURKi				
28	ANGIO	MET	RAF	Her	CDK 4_6	PI3K	ERK	WNT	JAK_STAT	DNA_REP	CTLA4			
29	MET	Antiap	RAS	DNA_RE P	CTLA4	RAF								
30	ANGIO	MEK	RAS	RAF	WNT	Her	CDK 4_6	mTKPT	DNA_REP	NOTCH				
31	Her	ERK	RAS	RAF	AGPT	MET	HEDGEH OG							
32	Her	AGPT	ANGIO	RAS	HDAC	HEDGEH OG								
33	Her	CDK 4_6	AURKA	PI3K	MEK	ERK	mTKPT	RAS	RAF	PARP	HDAC	JAK_STAT	PD1L	CTLA4
34	Her	PI3K	MET	JAK_STA T	CDK 4_6	RAS	RAF	TELOM	CTLA4					
35	Her	FGF	RAS	RAF	IGF_War	WNT	JAK_STAT	NOTCH	CDK 4_6	AGPT	PI3K	MEK	mTKPT	HDAC
36	Her	PI3K	MET	MEK	FGF	mTKPT	RAS	RAF	WNT	AGPT	HDAC	JAK_STAT	CTLA4	
37	Her	ANGIO	MET	MEK	ERK	FGF	mTKPT	RAF	IGF_War	HDAC	JAK_STAT	HEDGEH OG	AGPT	PI3K
38	ANGIO	AGPT	FGF	mTKPT	RAF	IGF_War	HEDGEH OG	CDK 4_6	AURKA	ERK	Antiap	PARP	DNA_REP	NOTCH
39	ANGIO	RAS	PD1L	Her	AGPT	AGPT								
40	Her	ANGIO	AGPT	MET	ERK	FGF	mTKPT	RAF	IGF_War	JAK_STAT	DNA_REP	CTLA4	PI3K	MEK
41	CDK 4_6	MET	ERK	Antiap	mTKPT	DNA_REP	CTLA4	AURKA	IGF_War	WNT	PARP			
42	Her	ANGIO	AGPT	PI3K	MET	RAF	CTLA4							
43	ANGIO	ERK	IGF_Wa r	WNT	Her	AURKA	TELOM	PARP	JAK_STAT	DNA_REP	PD1L			
44	Her	ANGIO	AGPT	PI3K	ERK	RAF	IGF_War	PARP	HEDGEH OG	PD1L				
45	Her	CDK 4_6	AGPT	PI3K	MET	MEK	mTKPT	RAF	WNT	JAK_STAT	NOTCH	RAS	IGF_War	PARP
46	Her	CDK 4_6	MET	MEK	RAF	JAK_STAT	ANGIO	PD1L	CTLA4					
47	ANGIO	AGPT	ERK	HDAC	Her									
48	Her	RAF	PI3K	NOTCH	ERK									
49	AGPT	MEK	RAF	CTLA4	ANGIO	MET	RAS	JAK_STAT	PD1L					
50	Her	CDK 4_6	MEK	PD1L	ANGIO	AGPT	FGF	mTKPT	IGF_War	WNT				
51	Her	CDK 4_6	AURKA	PI3K	MET	ERK	Antiap	RAS	RAF	HEDGEH OG	DNA_REP	NOTCH	PD1L	CTLA4
52	CDK 4_6	ANGIO	AGPT	PI3K	MET	FGF	RAS	NOTCH	PD1L	AURKA	mTKPT	TELOM	IGF_War	JAK_STAT
53	Her	ANGIO	AGPT	MET	ERK	RAS	IGF_War	WNT	RAF	PD1L	CTLA4			
54	Her	ERK	RAS	WNT	PARP	HDAC	HEDGEH	Antiap	TELOM	IGF_War	DNA_REP	PD1L		

							OG							
55	Her	AURKA	ANGIO	Antiap	RAS	CDK 4_6	FGF	mTKPT	TELOM	WNT	DNA_REP	CTLA4	CDK 4_6	FGF
56	AGPT	PI3K	MEK	RAF	JAK_STAT	CTLA4	CDK 4_6	Antiap	mTKPT	RAS	PARP	DNA_REP	PD1L	
57	Antiap	Her	AURKA	FGF	TELOM	IGF_War	PARP	DNA_REP	HDAC	HEDGE HOG				
58	Antiap	CDK 4_6	AURKA	PI3K	MET	WNT	PARP	HEDGE HOG	DNA_REP	NOTCH	mTKPT	TELOM	HDAC	
59	Antiap	CDK 4_6	AURKA	AGPT	PI3K	ERK	FGF	mTKPT	WNT	PARP	HDAC	HEDGEH OG	NOTCH	ANGIO
60	Antiap	Her	MET	FGF	RAS	RAF	WNT	CDK 4_6	PI3K	PARP	HDAC	JAK_STAT		
61	Antiap	CDK 4_6	AURKA	PI3K	ERK	FGF	RAS	TELOM	IGF_War	WNT	PARP	HDAC	HEDGEH OG	DNA_REP
62	Antiap	Her	CDK 4_6	AURKA	PI3K	MET	TELOM	WNT	PARP	HEDGEH OG	DNA_REP	NOTCH	MEK	FGF
63	Antiap	AURKA	MET	ERK	RAS	HDAC	JAK_STAT	HEDGEH OG	DNA_REP	NOTCH	CDK 4_6	RAF	IGF_War	WNT
64	Antiap	AURKA	ANGIO	AGPT	MEK	ERK	FGF	RAS	TELOM	IGF_War	JAK_STAT	HEDGEH OG	NOTCH	
65	Antiap	Her	PI3K	MET	MEK	FGF	mTKPT	RAF	TELOM	JAK_STAT	PD1L	AURKA	ERK	RAS
66	Antiap	Her	ANGIO	MET	ERK	FGF	mTKPT	TELOM	IGF_War	HDAC	NOTCH	AURKA	HEDGEH OG	DNA_REP
67	Antiap	CDK 4_6	AURKA	ANGIO	FGF	mTKPT	PARP	HDAC	DNA_REP	NOTCH	Her			
68	Antiap	AURKA i	ERK	FGF	mTKPT	TELOM	HEDGEH OG	DNA_REP	NOTCH	MET	RAS	WNT	PARP	
69	Antiap	MEK	ERK	RAF	IGF_War	HDAC	NOTCH	PD1L						
70	Antiap	AURKA	PI3K	ERK	mTKPT	RAF	PARP	HDAC	JAK_STAT	DNA_REP				
71	Antiap	CDK 4_6	CDK 4_6	AGPT	PI3K	MEK	ERK	mTKPT	TELOM	PARP	HDAC	DNA_REP	PD1L	ANGIO
72	Antiap	AGPT	ERK	FGF	RAS	TELOM	HDAC	HEDGEH OG	NOTCH	PARP				
73	Antiap	ANGIO	ERK	RAF	IGF_War	MEK	TELOM	NOTCH	PD1L					
74	Antiap	AURKA	ANGIO	PI3K	MET	mTKPT	RAS	HEDGEH OG	PD1L	CTLA4	Her	AGPT	FGF	WNT
75	Her	AURKA	ANGIO	AGPT	PI3K	Antiap	FGF	mTKPT	RAS	RAF	TELOM	WNT	HDAC	HEDGEH OG
76	JAK_ST AT	PI3K	ERK	Antiap	FGF	NOTCH	CTLA4							
77	PD1L	MEK	RAS	JAK_STA T										
78	ANGIO	AGPT	Antiap	RAS	RAF	IGF_War	WNT	NOTCH	PD1L	CTLA4	Her	AURKA	MEK	HDAC
79	Her	CDK 4_6	AURKA	Antiap	FGF	RAS	JAK_STAT	HEDGEH OG	DNAREP	CTLA4	ANGIO	MEK	ERK	mTKPT
80	Her	CDK 4_6	PI3K	MET	MEK	TELOM	IGF_War	HDAC	JAK_STAT	AURKA	ANGIO	AGPT	ERK	FGF
81	AURKA	MET	Antiap	PARP	HEDGEH OG	AGPT								
82	Her	MET	ERK	IGF_War	CTLA4									
83	ANGIO	AGPT	MET	ERK	mTKPT	CTLA4	CDK 4_6	MEK	FGF	RAF	IGF_War	DNAREP	PD1L	
84	CDK 4_6	ANGIO	AGPT	MET	FGF	mTKPT	TELOM	IGF_War	WNT	HEDGEH OG	NOTCH	PD1L	CTLA4	Her
85	CDK 4_6	ANGIO	AGPT	RAS	RAF	JAK_STAT	NOTCH	PD1L	CTLA4	PI3K	mTKPT			
86	AURKA	ANGIO	Antiap	FGF	IGF_War	WNT	HEDGEH OG	DNAREP	NOTCH	MET	RAF	PARP	PD1L	CTLA4
87	ANGIO	AGPT	ERK	Antiap	mTKPT	RAF	IGF_War	PARP	DNAREP	NOTCH	PD1L	Her	AURKA	FGF
88	PI3K	mTKPT	WNT	PARP	Her	AGPT	MEK	RAS	TELOM	HDAC	NOTCH	CTLA4		
89	CDK 4_6	FGF	RAS	RAF	IGF_War	WNT	HEDGEH OG	HDAC	NOTCH	PD1L				
90	AGPT	HEDGE HOG	ANGIO	PI3K	PI3K	MEK	FGF	mTKPT	WNT	HDAC				
91	AURKA	PI3K	WNT	JAK_STA T	HEDGEH OG	NOTCH	PD1L	Her	CDK 4_6	ERK	mTKPT	RAS	TELOM	PARP
92	AURKA	PI3K	TELOM	WNT	PARP	HEDGEH OG	HEDGEH OG	DNAREP	NOTCH	PD1L	Her	CDK 4_6	ANGIO	AGPT
93	Her	PI3K	Antiapo pt	TELOM	WNT	PARP	HEDGEH OG	DNAREP	NOTCH	PD1L	CTLA4	CDK 4_6	AGPT	HDAC
94	FGF	IGF_Wa r	HDAC	PI3K	MET	MEK	mTKPT	RAS	TELOM	PARP	JAK_STAT	HEDGEH OG	DNAREP	PD1L
95	Her	AURKA	AGPT	PI3K	Antiap	FGF	TELOM	IGF_War	PARP	HDAC	HEDGEH OG	NOTCH	ANGIO	MET
96	Her	AURKA	PI3K	MEK	mTKPT	TELOM	WNT	PARP	HDAC	HEDGEH OG	DNAREP	NOTCH	CDK 4_6	ANGIO
97	ERK	Antiap	RAS	JAK_STA T	HEDGEH OG	ERK	TELOM	PARP	DNAREP					
98	RAS	JAK_ST AT	AGPT	PI3K	MEK	ERK	RAF	PD1L						
99	ANGIO	AGPT	PI3K	MET	MEK	mTKPT	PD1L	CTLA4	FGF	RAS	NOTCH			
100	Her	CDK 4_6	AURKA	AGPT	MET	MEK	Antiap	FGF	mTKPT	RAF	TELOM	IGF_War	PARP	HDAC
101	Her	CDK 4_6	AURKA	PI3K	MEK	Antiap	FGF	RAS	JAK_STAT	HEDGEH OG	DNAREP	PD1L	CTLA4	RAF
102	JAK_ST AT	AGPT	ERK	Antiap	PD1L									
103	Her	CDK 4_6	AURKA	mTKPT	TELOM	IGF_War	PARP	HDAC	HEDGEH OG	DNAREP	PI3K	ERK		
104	ANGIO	RAS	CTLA4	RAF	JAK_STAT	HEDGEH OG	PD1L							
105	FGF	MEK	ERK	RAF	TELOM	IGF_War	WNT	HEDGEH OG	DNAREP	NOTCH				
106	CDK 4_6	AURKA	AGPT	ERK	Antiap	FGF	mTKPT	WNT	HEDGEH OG	DNAREP	CTLA4	Her	RAS	TELOM
107	MEK	IGF_Wa r	PARP	HDAC	JAK_STAT	DNAREP	PD1L	CTLA4	AURKA	ANGIO	ERK	Antiapopt	mTKPT	TELOM
108	CDK 4_6	AURKA	PI3K	Antiap	mTKPT	TELOM	HDAC	JAK_STAT	DNAREP	PD1L	CTLA4			
109	CDK 4_6	AURKA	PI3K	MEK	Antiap	mTKPT	TELOM	WNT	HDAC	DNAREP	NOTCH	PI3K	PARP	
110	FGF	IGF_Wa r	JAK_ST AT	PD1L	ERK	RAF	TELOM	HEDGEH OG						
111	ANGIO	TELOM	HDAC	NOTCH	MEK	ERK	mTKPT	IGF_War	HEDGEH OG	PD1L				
112	CDK 4_6	PI3K	Antiap	FGF	RAS	TELOM	IGF_War	WNT	PARP	HDAC	HEDGEH OG	DNAREP	NOTCH	Her
113	Her	AURKA	PI3K	ERK	Antiap	FGF	TELOM	PARP	HDAC	JAK_STAT	DNAREP	NOTCH	CTLA4	CDK 4_6

114	MEK	WNT	PARP	NOTCH	CTLA4	CDK 4_6	AURKA	ANGIO	PI3K	ERK	RAS	RAF	TELOM	IGF_War
115	ANGIO	AGPT	Antiap	WNT	PARP	HEDGEH OG	DNAREP	NOTCH	MEK	mTKPT	HDAC	PD1L		
116	ERK	Antiap	FGF	WNT	PARP	HDAC	HEDGEH OG	NOTCH	CDK 4_6					
117	AGPT	RAS	IGF_Wa r	PD1L	ANGIO	FGF	TELOM	WNT	PARP	HEDGEH OG	NOTCH			
118	Her	ERK	JAK_ST AT	PD1L	CTLA4	AGPT	PI3K	MET	MEK	FGF	RAF	IGF_War	PARP	HDAC
119	CDK 4_6	PI3K	Antiap	PARP	HDAC	HEDGEH OG	DNAREP	CTLA4	mTKPT	TELOM	WNT			
120	FGF	RAF	TELOM	WNT	PARP	JAK_STAT	PD1L	MEK	ERK	Antiapopt	mTKPT	IGF_War	NOTCH	
On t	AURKA	PI3K	MEK	Antiapop	RAS	TELOM	WNT	PARP	HDAC	JAK_STAT	HEDGEH OG	DNAREP	NOTCH	PD1L

Supplemental Table 7: Trends of cooactivation of interventional points. Codes used to designate interventional points are described in Table 1. Empty boxes are non-activated interventional points (Score≤5). Boxes displaying 1 are activated interventional points (Score >5)

CTLA4	PD1L	mek	mTor	pi3k	ERK	met	AurkA	cdk4,6	HER	Angio	FGF	PARP	Ras/RAF	IGF	DNAREP	mtor/PI3K	Histo
1	1	1										1	1	1	1		AC_mP53
1	1			1				1					1	1	1	1	AC_mP53
	1		1							1		1	1			1	AC_mP53
1	1	1					1	1	1	1			1		1		AC_mP53
1	1	1	1	1			1		1	1			1		1	1	AC_mP53
	1								1				1	1			AC_mP53
	1	1	1	1	1	1		1	1	1			1	1		1	AC_mP53
	1	1	1		1						1		1			1	AC_wtp53
1	1	1				1	1	1					1				AC_wtp53
1	1					1	1		1		1		1		1		AC_wtp53
1	1	1	1	1		1	1	1	1	1	1				1	1	AC_wtp53
1	1	1	1		1		1	1	1			1	1			1	AC_wtp53
1	1	1	1	1		1	1	1	1	1			1		1	1	AC_wtp53
1	1	1	1	1	1	1						1	1			1	AC_wtp53
1	1					1				1			1	1			AC_wtp53
1	1			1		1	1		1	1			1	1		1	AC_wtp53
1	1	1	1	1	1		1	1	1			1	1			1	AC_wtp53
	1								1	1			1				AC_wtp53
	1				1		1		1	1		1		1	1		AC_wtp53
	1			1	1				1	1		1	1	1		1	AC_wtp53
1	1	1				1		1	1				1				AC_wtp53
1	1	1				1				1			1				AC_wtp53
	1	1	1					1	1	1							AC_wtp53
1	1			1	1	1	1	1	1		1	1	1	1	1	1	AC_wtp53
	1		1	1		1	1	1		1	1		1			1	AC_wtp53
1	1				1	1			1	1			1	1		1	AC_wtp53
	1				1				1				1	1	1	1	AC_wtp53
1	1	1	1	1				1					1		1	1	AC_wtp53
	1			1	1	1	1	1			1	1	1		1	1	SCC_mp53
	1	1	1	1	1	1	1		1		1		1	1	1	1	SCC_mp53
	1	1			1								1	1		1	SCC_mp53
	1	1	1	1	1			1		1	1	1		1	1	1	LCC_mp53
	1	1			1					1			1	1		1	LCC_mp53
1	1		1	1		1	1		1	1	1		1		1	1	SCC_mp53
1	1		1	1	1	1	1	1	1	1	1	1	1		1	1	SCC_wtp53
	1	1											1				SCC_wtp53
1	1	1					1		1	1			1	1			LCC_wtp53
1	1	1	1		1	1		1		1	1		1	1	1	1	LCC_wtp53
1	1	1	1	1		1	1	1	1	1	1			1	1	1	SCC_wtp53
1	1		1	1				1		1			1			1	SCC_wtp53
1	1					1	1			1			1				SCC_wtp53
	1		1		1		1		1	1	1		1	1	1	1	SCC_wtp53
	1							1			1		1				LCC_wtp53
1	1		1	1	1		1	1	1			1	1		1	1	SCC_wtp53
1	1					1	1	1	1	1		1			1		SCC_wtp53
1	1			1				1	1			1			1	1	SCC_wtp53
1	1	1	1			1					1	1	1	1	1		SCC_wtp53
	1	1		1	1								1			1	SCC_wtp53
1	1	1	1	1		1				1	1		1			1	SCC_wtp53
1	1	1	1			1	1	1	1		1	1	1	1			LCC_wtp53
1	1	1		1			1	1	1			1	1		1	1	SCC_wtp53
	1																SCC_wtp53
	1									1			1				SCC_wtp53
1	1	1	1		1		1			1			1	1	1	1	LCC_wtp53
1	1		1	1			1	1							1	1	SCC_wtp53
	1				1						1		1	1		1	SCC_wtp53
	1	1	1		1					1				1		1	SCC_wtp53
1	1	1	1	1	1		1	1	1		1	1			1	1	SCC_wtp53
	1	1	1		1					1					1	1	SCC_wtp53
	1									1	1	1	1	1			SCC_wtp53
1	1	1		1	1	1		1	1		1		1	1		1	SCC_wtp53
	1	1	1		1						1	1	1	1		1	SCC_wtp53

1	1	1		1			1				1	1	1		1	1	SCC_wtp53
1		1				1		1	1	1			1				AC_wtp53
1		1							1	1	1		1	1			AC_wtp53
1		1						1		1							AC_wtp53
1				1	1	1		1	1	1			1		1	1	AC_wtp53
1						1							1		1		AC_wtp53
1				1		1		1	1			1	1			1	AC_wtp53
1		1	1	1		1			1				1			1	AC_wtp53
1		1			1	1			1	1	1		1	1			AC_wtp53
1		1	1	1	1	1			1	1	1		1	1	1	1	AC_wtp53
1			1		1	1	1	1				1		1	1	1	AC_wtp53
1				1		1			1	1			1			1	AC_wtp53
1		1	1	1		1		1	1				1	1		1	AC_wtp53
1				1	1	1	1	1	1		1	1	1		1	1	AC_wtp53
1			1				1	1	1	1	1		1			1	AC_wtp53
1					1	1	1	1				1	1		1		SCC_mp53
1			1		1	1	1	1	1	1	1		1		1	1	SCC_wtp53
1				1	1						1					1	SCC_wtp53
1		1	1		1		1	1	1	1	1		1		1	1	SCC_wtp53
1		1		1	1	1	1	1	1	1	1			1		1	SCC_wtp53
1					1	1			1					1			LCC_wtp53
1		1	1	1		1			1			1	1			1	SCC_wtp53
1			1		1		1	1	1		1	1	1		1	1	SCC_wtp53
1		1		1	1		1	1		1		1	1			1	SCC_wtp53
1			1	1				1				1			1	1	SCC_wtp53
			1	1	1		1	1			1	1	1	1	1	1	AC_mP53
			1		1	1		1					1			1	AC_mP53
			1				1	1	1	1						1	AC_mP53
								1	1	1			1	1			AC_mP53
									1			1					AC_wtp53
			1						1		1		1	1		1	AC_wtp53
				1	1	1			1							1	AC_wtp53
			1				1			1					1	1	AC_wtp53
		1	1					1	1	1			1		1	1	AC_wtp53
					1	1			1				1				AC_wtp53
									1	1			1				AC_wtp53
		1	1	1				1	1		1		1	1		1	AC_wtp53
			1		1		1	1		1		1	1	1	1	1	AC_wtp53
					1				1	1							AC_wtp53
				1	1				1				1			1	AC_wtp53
							1		1		1	1		1	1		SCC_mp53
			1	1		1	1	1				1			1	1	SCC_mp53
		1	1	1	1	1	1	1		1	1		1			1	SCC_mp53
				1		1		1	1			1	1			1	SCC_mp53
		1	1	1		1	1	1	1		1	1			1	1	SCC_mp53
		1			1		1			1	1		1	1			SCC_mp53
			1		1	1	1		1	1	1				1	1	SCC_mp53
			1				1	1	1		1	1			1	1	SCC_mp53
			1		1		1				1	1	1		1	1	SCC_mp53
			1	1	1		1					1	1		1	1	SCC_mp53
												1					SCC_mp53
					1	1											LCC_wtp53
		1	1	1							1					1	SCC_wtp53
				1		1	1		1	1		1	1			1	SCC_wtp53
		1	1	1			1	1	1	1	1	1			1	1	SCC_wtp53
					1							1	1		1		SCC_wtp53
			1	1	1		1	1	1			1		1	1	1	LCC_wtp53
		1			1								1	1	1		SCC_wtp53
			1	1			1	1				1			1	1	LCC_wtp53
				1	1		1	1	1		1	1	1	1	1	1	SCC_wtp53
								1			1	1					SCC_wtp53
CTLA4	PD1L	mek	mTor	pi3k	ERK	met	AurkA	cdk4,6	HER	Angio	FGF	PARP	Ras/RAF	IGF	DNAREP	mtor/PI3K	Histo
**61**	**63**	**54**	**59**	**55**	**57**	**51**	**55**	**60**	**68**	**56**	**47**	**47**	**88**	**44**	**56**	**83**	**123**
**50**	**51**	**44**	**48**	**45**	**46**	**41**	**45**	**49**	**55**	**46**	**38**	**38**	**72**	**36**	**46**	**67**	**100**

Table 8: *Selection of the most frequent combinations, taking into account trends of coactivation. For each of the first and second drugs, number of patients (upper case) and % (lower case) are shown. For each of the third drugs, number of patients out of 121 and % are shown*

First drug	NB/%	Second drug	NB/%	Third drug	Nb	%
RAS/RAF	88	mTor/PI3K	60	PD1L	34	28
				CTLA4	33	27
				CDK4,6	32	26
				AURKA	29	24
				DNARepair	28	23
	72%		49%	ANGIO	27	22
				MET	27	22
				FGF	26	21
				PARP	24	20
				IGF	23	19
RAS/RAF	88	MET	40	CTLA4	32	26
				mTor/PI3K	27	22
				PD1L	22	18
				ANGIO	20	16
				CDK4,6	21	17
	72%		33%	AURKA	17	14
				FGF	17	14
				DNARepair	15	12
				IGF	13	11
				PARP	12	10
RAS/RAF	88	CDK4,6	40	mTor/PI3K	32	26
				CTLA4	27	22
				AURKA	23	19
				MET	21	17
				PD1L	20	16
	72%		33%	DNARepair	20	16
				ANGIO	17	14
				FGF	17	14
				PARP	17	14
				IGF	12	10
mTor/PI3K	83	RAS/RAF	60	PD1L	34	28
				CTLA4	33	27
				CDK4,6	32	26
				AURKA	29	24
				DNARepair	28	23
	67%		49%	MET	27	22
				ANGIO	27	22
				FGF	26	21
				PARP	24	20
				IGF	23	19
CDK4,6	63	RAS/RAF	51	mTor/PI3K	34	28
				CTLA4	27	22
				ANGIO	24	20
				IGF	23	19
				MET	22	18
	51%		41%	AURKA	22	18
				CDK4,6	20	16
				PD1L	20	16
				FGF	19	15
				PARP	16	13

PD1L	63	mTor/PI3K	42	RAS/RAF	34	28
				CTLA4	25	20
				DNARepair	23	19
				CDK4,6	21	17
				ANGIO	21	17
	51%		34%	AURKA	20	16
				IGF	19	15
				FGF	18	15
				MET	16	13
				PARP	15	12
MEK	54	RAS/RAF	42	CTLA4	29	24
				PD1L	28	23
				mTor/PI3K	28	23
				CDK4,6	19	15
				ANGIO	19	15
	44%		34%	IGF	19	15
				AURKA	16	13
				FGF	16	13
				DNARepair	15	12
				parp	11	9
CDK4,6	60	mTor/PI3K	48	RAS/RAF	32	26
				AURKA	32	26
				DNARepair	32	26
				CTLA4	29	24
				parp	26	21
	49%		39%	FGF	23	19
				MET	22	18
				PD1L	21	17
				ANGIO	20	16
				IGF	15	12
MET	51	RAS/RAF	40	CTLA4	32	26
				mTor/PI3K	27	22
				PD1L	22	18
				ANGIO	21	17
				MEK	19	15
	41%		33%	AURKA	17	14
				FGF	17	14
				DNARepair	15	12
				IGF	13	11
				PARP	12	10
ANGIO	56	RAS/RAF	41	mTor/PI3K	27	22
				PD1L	24	20
				MET	20	16
				MEK	19	15
				AURKA	19	15
	46%		33%	IGF	17	14
				CDK4,6	16	13
				FGF	15	12
				DNARepair	14	11
				PARP	7	6
